# Associations of sarcopenia and sarcopenic obesity with hypertension: a systematic review and meta-analysis

**DOI:** 10.3389/fnut.2026.1881526

**Published:** 2026-09-07

**Authors:** Xiang Li, Yannan Zhao, Jinqing Liu, Zexing Wen, Yujian Chen, Wei Xie

**Affiliations:** 1 School of Leisure and Sports, Chengdu Sport University, Chengdu, China; 2 School of Acupuncture and Tuina, Chengdu University of Traditional Chinese Medicine, Chengdu, China; 3 School of Leisure and Sports, Ginkgo College of Hospitality Management, Chengdu, China; 4 School of Physical Education and Health, Chengdu University of Traditional Chinese Medicine, Chengdu, China

**Keywords:** body composition, hypertension, meta-analysis, sarcopenia, sarcopenic obesity

## Abstract

**Background:**

Sarcopenia and sarcopenic obesity have been increasingly investigated in relation to hypertension, but observational findings remain inconsistent. This systematic review and meta-analysis aimed to quantitatively synthesize the associations of sarcopenia and sarcopenic obesity with hypertension in adults.

**Methods:**

PubMed, Embase, the Cochrane Library, and Web of Science were searched from inception to July 20, 2026. Observational studies reporting associations of sarcopenia or sarcopenic obesity with hypertension were included. Effect estimates were pooled using random-effects restricted maximum likelihood models. Subgroup analyses were conducted according to study design, age, sarcopenia diagnostic criteria, and hypertension definition. Exploratory stratified analyses were additionally performed according to effect measure type [odds ratio (OR) versus hazard ratio (HR)]. The protocol was registered in PROSPERO (CRD420261393787).

**Results:**

Nine studies involving 346,748 participants were included. Sarcopenia was significantly associated with hypertension (pooled relative effect estimate: 1.45, 95% CI: 1.08–1.94; *p* = 0.01; I^2^ = 95.51%). Sarcopenic obesity was also significantly associated with hypertension (pooled relative effect estimate: 2.49, 95% CI: 1.75–3.56; *p* < 0.001; I^2^ = 86.85%). Subgroup analyses generally showed associations in the same direction, although most tests for subgroup differences were not statistically significant. Exploratory analyses stratified by effect measure type did not identify statistically significant subgroup differences for sarcopenia (*p* = 0.10) or sarcopenic obesity (*p* = 0.26), although the HR strata were based on a limited number of studies. Leave-one-out sensitivity analyses retained the direction and statistical significance of the pooled associations. Because of the limited number of studies, publication bias assessments were considered exploratory.

**Conclusion:**

Sarcopenia and sarcopenic obesity were both significantly associated with hypertension. However, substantial heterogeneity and the observational nature of the evidence warrant cautious interpretation and preclude causal inference. Further prospective studies using harmonized definitions and diverse populations are needed to clarify temporality, sources of heterogeneity, and potential clinical implications.

**Systematic review registration:**

https://www.crd.york.ac.uk/PROSPERO/view/CRD420261393787, identifier CRD420261393787.

## 1 Introduction

With advances in healthcare and increasing life expectancy, population aging has become a major global demographic trend. Aging is accompanied by progressive changes in the cardiovascular, musculoskeletal, and metabolic systems, contributing to an increasing burden of chronic diseases and age-related syndromes (1). Recent community-based evidence further indicates that adverse body-composition and fat-distribution profiles in older adults are associated with both hypertension and dysmobility syndrome, highlighting the interconnected cardiovascular, musculoskeletal, and metabolic consequences of aging (2, 3). However, cardiometabolic disorders are not confined to older populations. Hypertension, characterized by sustained elevation of systolic and/or diastolic blood pressure, remains a major preventable contributor to morbidity and mortality worldwide (4, 5). It is strongly associated with adverse cardiovascular and cerebrovascular outcomes and continues to impose a substantial burden on individuals and healthcare systems (6). Although lifestyle modification and pharmacological treatment remain central to hypertension management, blood pressure control remains suboptimal in many affected individuals because of medication non-adherence, adverse effects, clinical inertia, and healthcare-related barriers (7, 8).

Increasing attention has been directed toward the relationship between body composition and cardiovascular health. Sarcopenia is a progressive skeletal muscle disorder characterized primarily by reduced muscle strength, together with reduced muscle quantity or quality and, in more severe cases, impaired physical performance (9, 10). Sarcopenic obesity refers to the coexistence of sarcopenia and obesity and represents a distinct phenotype characterized by impaired muscle health in the presence of excess adiposity (11). Although sarcopenia has traditionally been studied mainly in older adults, impaired muscle health and unfavorable body-composition profiles may also occur earlier in adulthood in association with physical inactivity, poor diet quality, obesity, and metabolic dysfunction. Skeletal muscle and adipose tissue participate in metabolic, endocrine, and inflammatory regulation, and alterations in glucose utilization, myokine signaling, insulin sensitivity, oxidative stress, and vascular homeostasis may provide biologically plausible links between these body-composition phenotypes and hypertension (12–14). However, these pathways remain potential explanations rather than mechanisms established by the available observational evidence.

Epidemiological studies examining the associations of sarcopenia and sarcopenic obesity with hypertension have reported inconsistent findings (15). Bai et al. previously synthesized evidence on sarcopenia and hypertension specifically in older adults, but sarcopenic obesity was not separately evaluated within that review (16). Since then, additional studies involving broader adult age ranges and different body-composition phenotypes have become available. Considerable variation also exists across studies in age structure, study design, exposure definitions, muscle assessment methods, and hypertension ascertainment, limiting direct comparison and potentially contributing to differences in reported estimates.

Therefore, this systematic review and meta-analysis aimed to synthesize the available observational evidence on the associations of sarcopenia and sarcopenic obesity with hypertension among adults aged ≥18 years. Sarcopenia and sarcopenic obesity were evaluated separately, and potential sources of between-study heterogeneity were explored according to study design, age, sarcopenia diagnostic criteria, and hypertension definition. By extending previous evidence beyond sarcopenia in older adults and incorporating sarcopenic obesity within the same systematic framework, this study aimed to provide a broader quantitative assessment of the relationship between muscle–adiposity phenotypes and hypertension.

## 2 Methods

### 2.1 Search strategy

The protocol for this systematic review and meta-analysis was prospectively registered in the PROSPERO database (CRD420261393787). This study was conducted and reported in accordance with the Preferred Reporting Items for Systematic Reviews and Meta-Analyses (PRISMA) guidelines (17, 18).

An updated comprehensive literature search was conducted by two independent reviewers (XL and YNZ) in PubMed, Embase, the Cochrane Library, and Web of Science from database inception to July 20, 2026. The search strategy combined controlled vocabulary terms, including Medical Subject Headings (MeSH) in PubMed and Emtree terms in Embase, with free-text terms. The search was constructed using two main concept blocks: (1) sarcopenia or sarcopenic obesity, and (2) hypertension or elevated blood pressure. Terms within each concept block were combined using the Boolean operator “OR,” and the two concept blocks were then combined using “AND.”

The sarcopenia-related search terms included “Sarcopenia,” “Sarcopenias,” “muscle weakness,” “low muscle mass,” “decreased muscle mass,” “muscle strength,” “muscle loss,” “muscle depletion,” “muscular atrophy,” and “sarcopenic obesity.” The hypertension-related search terms included “Hypertension,” “Blood Pressure, High,” “Blood Pressures, High,” “High Blood Pressure,” “High Blood Pressures,” “elevated blood pressure,” and “arterial hypertension.”

The PubMed search strategy was as follows: (Sarcopenia[MeSH Terms] OR “Sarcopenias”[Title/Abstract] OR “Muscle weakness”[Title/Abstract] OR “Low muscle mass”[Title/Abstract] OR “Decreased muscle mass”[Title/Abstract] OR “Muscle strength”[Title/Abstract] OR “Muscle loss”[Title/Abstract] OR “Muscle depletion”[Title/Abstract] OR “Muscular atrophy”[Title/Abstract] OR “sarcopenic obesity”[MeSH Terms]) AND (Hypertension[MeSH Terms] OR “Blood Pressure, High”[Title/Abstract] OR “Blood Pressures, High”[Title/Abstract] OR “High Blood Pressure”[Title/Abstract] OR “High Blood Pressures”[Title/Abstract] OR “Elevated blood pressure”[Title/Abstract] OR “Arterial hypertension”[Title/Abstract]).

**Figure 1:**
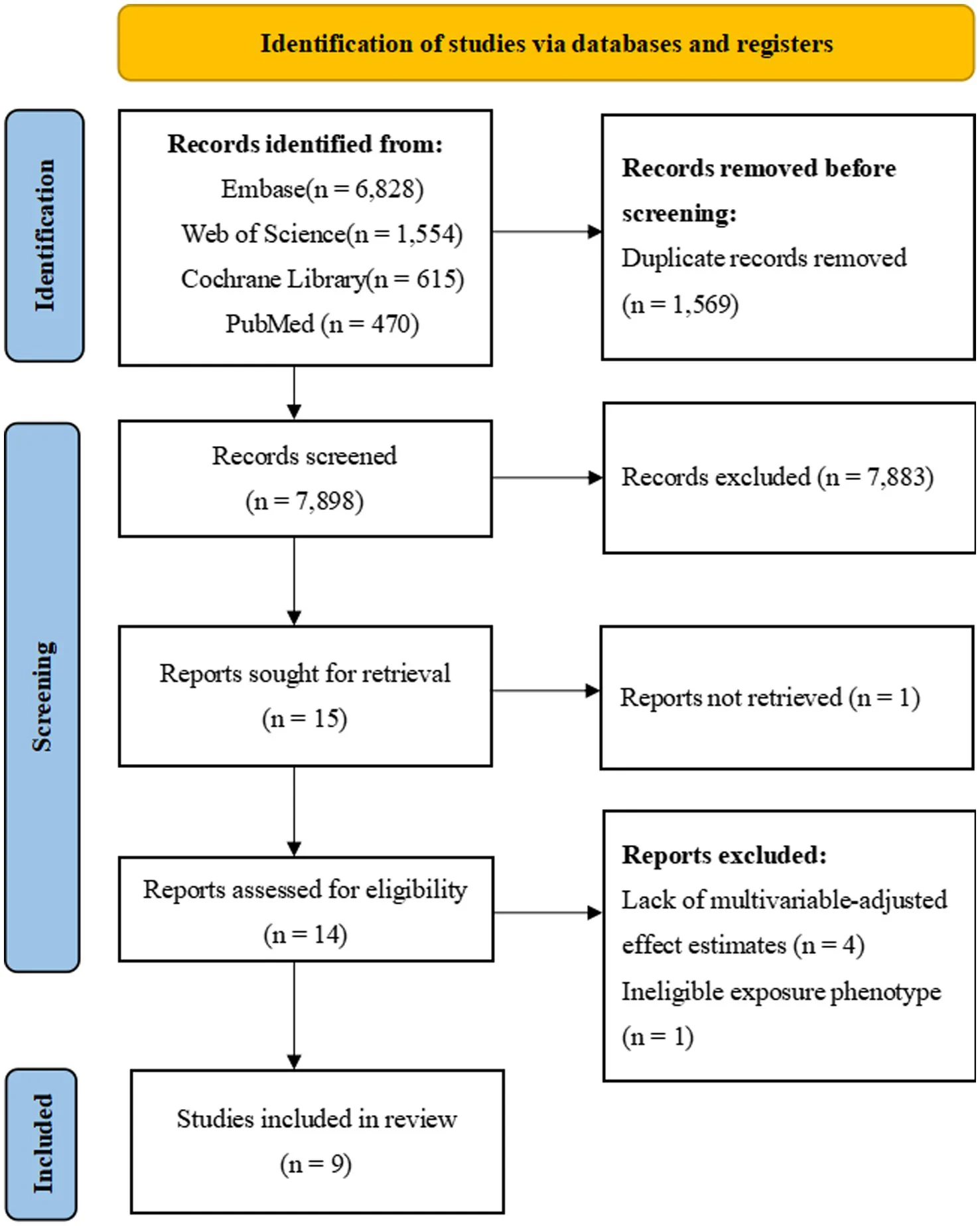
Literature screening flow chart.

The search strategies were adapted for Embase, the Cochrane Library, and Web of Science according to the syntax and indexing structure of each database. No restrictions were imposed on language or publication year during the electronic database search. The complete database-specific search strategies are provided in Supplementary Table S1. In addition, the reference lists of the included studies and relevant reviews were manually screened to identify any additional eligible studies. The detailed study selection process is shown in Figure 1.

### 2.2 Inclusion and exclusion criteria

The inclusion criteria were formulated according to the PECOS framework as follows: (1) Population: Adults aged ≥18 years, with no restrictions on sex, ethnicity, or geographic region. (2) Exposure: The exposures of interest were sarcopenia and/or sarcopenic obesity. Sarcopenia was defined according to the diagnostic criteria used in each original study, including internationally or regionally recognized consensus criteria, such as the Asian Working Group for Sarcopenia (AWGS), the European Working Group on Sarcopenia in Older People (EWGSOP), the Foundation for the National Institutes of Health (FNIH) Sarcopenia Project, or other explicitly reported study-specific definitions (9, 10, 19). These definitions generally involved one or more components of muscle mass, muscle strength, and/or physical performance. Sarcopenic obesity was defined as the coexistence of sarcopenia and obesity according to the criteria adopted in each included study (11). Because obesity definitions vary across populations, sex, ethnicity, and clinical guidelines, we accepted the obesity indicators and thresholds reported by the original studies, including body mass index (BMI), waist circumference, body fat percentage, fat mass, or other body-composition-based criteria. The population-specific nature of these thresholds was considered during data extraction and interpretation. (3) Comparator: The comparison group consisted of participants without sarcopenia or sarcopenic obesity, as defined in each original study. When available, the comparator was based not only on body composition parameters but also on the absence of impaired muscle strength or physical performance according to the corresponding diagnostic framework. (4) Outcome: The primary outcome was hypertension. Hypertension was defined according to the criteria used in each original study, including measured systolic and/or diastolic blood pressure thresholds, self-reported physician diagnosis, medical history of hypertension, use of antihypertensive medication, or diagnostic codes. Given the historical and regional variability in hypertension guidelines, studies using alternative but clearly reported diagnostic thresholds were considered eligible (5, 20). (5) Studies were required to report at least one multivariable-adjusted effect estimate for the association between sarcopenia or sarcopenic obesity and hypertension, including an adjusted odds ratio (OR) or adjusted hazard ratio (HR), together with its corresponding 95% confidence interval (CI). When multiple multivariable models were reported, the most fully adjusted estimate was extracted.

Studies were excluded if they met any of the following criteria: (1) reviews, systematic reviews, meta-analyses, editorials, conference abstracts, case reports, animal studies, or *in vitro* studies; (2) studies without extractable multivariable-adjusted ORs or HRs with corresponding 95% CIs; (3) studies that did not clearly report the diagnostic criteria for sarcopenia, sarcopenic obesity, or hypertension; (4) studies in which the exposure of interest was not sarcopenia or sarcopenic obesity, including studies evaluating osteosarcopenic obesity without separately extractable data for sarcopenic obesity; (5) studies based on duplicate or overlapping populations, in which case the study with the largest sample size, longest follow-up, or most comprehensive data was retained.

### 2.3 Literature screening

Following the automated removal of duplicate records using NoteExpress software, two independent reviewers performed a two-stage screening process. In the initial stage, the titles and abstracts of the identified records were screened to determine their potential eligibility. Subsequently, the full texts of the remaining studies were retrieved and independently evaluated against the predefined PRISMA-compliant inclusion and exclusion criteria. Any discrepancies between the reviewers were resolved through consensus or consultation with a third investigator.

### 2.4 Data extraction

Data extraction was performed independently by two reviewers (XL and YNZ) using a predesigned standardized data extraction form. The extracted information included the first author, publication year, country or region, study design, data source, participant characteristics, total sample size, sample size available for each exposure-specific analysis, follow-up duration for cohort studies, diagnostic criteria for sarcopenia, obesity, sarcopenic obesity, and hypertension, muscle mass assessment method, muscle mass adjustment index, exposure and comparator definitions, effect estimate type, effect estimates with 95% confidence intervals, and covariates adjusted for in the multivariable models.

Only multivariable-adjusted effect estimates were extracted for the primary meta-analyses. When multiple adjusted models were reported, the estimate from the model with the most comprehensive adjustment for demographic, behavioral, and clinical confounders was selected. For studies reporting results separately by sex or other independent subgroups, the subgroup-specific estimates were extracted as independent datasets when appropriate. If a study reported effect estimates for both sarcopenia and sarcopenic obesity, data for each exposure were extracted separately. Any discrepancies during data extraction were resolved through discussion, and a third reviewer (WX) was consulted when necessary.

### 2.5 Quality assessment

The methodological quality of the included studies was independently assessed by two reviewers (XL and YNZ) using study-design-specific versions of the Newcastle–Ottawa Scale (NOS) (21, 22). Prospective cohort studies were evaluated using the conventional NOS for cohort studies, which assesses three domains: selection of study groups, comparability of groups, and outcome assessment. The maximum score for cohort studies was 9 stars.

For cross-sectional studies, a modified NOS adapted for cross-sectional designs was used. This modified tool assessed three domains: selection of participants, comparability of study groups, and outcome/statistical assessment. The selection domain included representativeness of the sample, sample size, non-respondents, and ascertainment of exposure. The comparability domain evaluated whether important confounding factors were controlled for in the design or analysis. The outcome/statistical assessment domain evaluated outcome assessment and the appropriateness of the statistical test. The maximum score for cross-sectional studies was 10 stars.

Because the maximum possible score differed between study designs, quality ratings were interpreted within each study-design-specific scale. Studies were classified as high quality if they scored ≥7, moderate quality if they scored 5–6, and low quality if they scored ≤4. Any disagreements between the two reviewers were resolved through discussion, and a third reviewer (WX) was consulted when necessary. The detailed item-level quality assessment results are presented in Tables 1, 2.

**Table 1:** Quality assessment of prospective cohort studies using the original NOS.

Study	Items	Han et al. (30)	Du et al. (28)	Qiu et al. (27)	Zou et al. (25)
Selection of population	Representativeness of the exposed cohort	/	*	*	/
	Selection of the non-exposed cohort	*	*	*	*
	Ascertainment of exposure	*	*	*	*
	Demonstration that outcome of interest was not present at start of study	*	*	*	*
Comparability of populations	Comparability of cohorts on the basis of the design or analysis	**	**	**	**
Exposure/outcome assessment	Assessment of outcome	*	/	/	*
	Was follow-up long enough for outcomes to occur	*	*	*	*
	Adequacy of follow-up of cohorts	*	/	*	*
Score		8	7	8	8

**Table 2:** Quality assessment of cross-sectional studies using the modified NOS.

Study	Items	Park et al. (33)	Han et al. (32)	Kreidieh et al. (31)	Pasdar et al. (29)	Kim et al. (28)
Selection of population	Representativeness of the sample	*	*	/	*	*
	Sample size	*	*	/	*	*
	Non-respondents	*	/	/	/	/
	Ascertainment of exposure	**	**	**	**	**
Comparability of populations	Comparability of subjects on the basis of the design or analysis	**	**	*	**	**
Exposure/outcome assessment	Assessment of outcome	**	**	*	**	**
	Statistical test	*	*	*	*	*
Score		10	9	5	9	9

### 2.6 Statistical analysis

The association between sarcopenia or sarcopenic obesity and hypertension was evaluated using the effect estimates reported in the included studies. Odds ratios (ORs) and hazard ratios (HRs), together with their corresponding 95% confidence intervals (CIs), were extracted. Only multivariable-adjusted effect estimates were included in the primary meta-analyses. When multiple adjusted models were reported, the estimate from the model with the most comprehensive adjustment for potential confounders was selected.

All effect estimates were transformed to the natural logarithmic scale before pooling, and standard errors were calculated from the corresponding 95% CIs. Given the anticipated clinical and methodological heterogeneity across studies, including differences in study design, population characteristics, diagnostic criteria, muscle mass adjustment indices, and hypertension definitions, random-effects models were used for the main meta-analyses. The restricted maximum likelihood (REML) method was applied to estimate between-study variance.

Adjusted ORs and HRs were pooled on the logarithmic scale in the primary analyses and summarized as pooled relative effect estimates. To examine the potential influence of effect-measure heterogeneity, exploratory stratified analyses were conducted according to effect measure type (OR versus HR), separately for sarcopenia and sarcopenic obesity. Because the number of HR-reporting studies was limited and effect measure type partly overlapped with study design and outcome timing, these analyses were considered exploratory and interpreted cautiously. The combined estimates were described as associations rather than causal effects.

Statistical heterogeneity was assessed using Cochran’s Q test, the I^2^ statistic, and the between-study variance τ^2^. I^2^ values of approximately 25, 50, and 75% were considered to represent low, moderate, and high heterogeneity, respectively (23). To explore potential sources of heterogeneity, subgroup analyses were performed according to study design, age, sarcopenia diagnostic criteria, and hypertension definition, where data permitted. Tests for subgroup differences were performed, but the results were interpreted cautiously because of the limited number of studies in each subgroup.

Sensitivity analyses were conducted using a leave-one-out approach, in which each study or independent dataset was sequentially excluded and the pooled estimate was recalculated. In addition, to assess the consistency of the findings in longitudinal study designs, design-restricted sensitivity analyses were conducted by excluding cross-sectional studies and pooling only prospective cohort studies separately for sarcopenia and sarcopenic obesity, using the same random-effects REML approach as in the primary analyses.

Funnel plots and Egger’s regression tests were used to explore potential publication bias and small-study effects. Because fewer than 10 studies contributed to each exposure-specific meta-analysis, these assessments were considered exploratory and were interpreted cautiously because of their limited statistical power (24). The absence of statistically significant asymmetry was not interpreted as evidence that publication bias was absent.

All statistical analyses were performed using Stata software, version 17.0. A two-sided *p* value < 0.05 was considered statistically significant, except for Cochran’s Q test, for which *p* < 0.10 was considered to indicate statistically significant heterogeneity.

## 3 Results

### 3.1 Literature screening process and results

The updated electronic database search identified a total of 9,467 records from Embase (*n* = 6,828), Web of Science (*n* = 1,554), Cochrane Library (*n* = 615), and the PubMed (*n* = 470). After the removal of 1,569 duplicate records, 7,898 records remained for title and abstract screening. Of these, 7,883 records were excluded because they were clearly irrelevant, did not investigate sarcopenia or sarcopenic obesity, did not report hypertension as an outcome, or were not eligible study designs. A total of 15 reports were sought for retrieval, of which one could not be retrieved. The remaining 14 full-text reports were assessed for eligibility. Five reports were subsequently excluded: four because multivariable-adjusted effect estimates were unavailable and one because the exposure phenotype did not meet the predefined eligibility criteria. Finally, nine studies were included in the systematic review and meta-analysis (25–33).

### 3.2 Basic characteristics of the included literature

The basic characteristics of the included studies are summarized in Table 3. A total of 9 observational studies involving 346,748 participants were included in this systematic review and meta-analysis. The sample sizes of the included studies ranged from 184 to 183,091 participants. The mean age of participants ranged from 33.26 to 70.6 years across studies.

**Table 3:** Basic characteristics of the included studies.

Author	Year	Country	Study design	Age (years)	Follow-up (years)	Diagnostic criteria	Effect estimate (95% CI)	Sample size	Confounders adjusted	NOS scores
Park e et al.	2013	Korea	Cross-sectional study	50.1 ± 17.8	N/A	Sarcopenia: Appendicular skeletal muscle mass divided by weight (ASM/Wt) below two standard deviations from the mean of a sample of healthy young adults. Cut-off points: 29.5% for men and 23.2% for women. Sarcopenic Obesity: A combination of sarcopenia and obesity according to each definition (determined by high WC and low muscle mass values). Hypertension: Systolic blood pressure >140 mm Hg, diastolic blood pressure >90 mm Hg, or the self-reported current use of an antihypertensive medication.	OR (95% CI) for Sarcopenia and hypertension: 1.20 (0.61–2.36); OR (95% CI) for Sarcopenic obesity and hypertension: 2.91 (1.67–5.05).	6,832	Adjusted for age, sex, physical activity, alcohol consumption, smoking, diabetes, dyslipidemia and cardiovascular disease.	10
Han et al.	2014	Korea	Cross-sectional study	70.6 ± 0.3	N/A	Sarcopenia: Appendicular skeletal muscle mass divided by body weight (ASM/Wt) that was < 1 SD below the sex-specific mean for healthy young adults (aged 20–40). Sarcopenic Obesity: Presence of both obesity and sarcopenia based on the definitions above. Hypertension: Systolic BP (SBP) ≥ 140 mmHg, diastolic BP (DBP) ≥ 90 mmHg, or self-reported current use of antihypertensive medications.	OR (95% CI) for Sarcopenia and hypertension: 1.33 (1.06–1.67); OR (95% CI) for Sarcopenic obesity and hypertension: 1.89 (1.42–2.51).	4,846	Adjusted for age, sex, regular activity, current smoking, alcohol use, previous CHD, stroke, metabolic risk factors (fasting glucose, triglycerides, HDL cholesterol), body weight, waist circumference and dietary sodium & potassium intake.	9
Kreidieh et al.	2018	Lebanon	Cross-sectional study	33.26 ± 14.65	N/A	Sarcopenic Obesity: Based on the FNIH Sarcopenia Project criteria: Appendicular Lean Mass (ALM) divided by BMI (ALM/BMI) < 0.512 for women. Hypertension: Based on self-reported diagnosis.	OR (95% CI) for Sarcopenic obesity and hypertension: 4.48 (1.16–17.38).	184	Adjusting for a sedentary lifestyle, fast-food consumption, and smoking.	5
Han et al.	2020	Korea	Prospective cohort study	Men: 38.2 ± 6.6; Women: 37.1 ± 6.7	4 years	Sarcopenia: A muscle mass value less than one standard deviation (SD) from the mean value in a young reference population (i.e., individuals aged 19–39 years). Hypertension: An SBP ≥ 140 mmHg, a DBP ≥ 90 mmHg, a history of hypertension, or the use of antihypertensive medications.	Male: OR (95% CI) for Sarcopenia and hypertension: 1.46 (1.30–1.63); Female: OR (95% CI) for Sarcopenia and hypertension: 0.97 (0.76–1.23).	Men: 72,560 Women: 59,764	Adjusted for age, BMI, education level, SBP, diabetes, dyslipidemia, eGFR, HOMA-IR, hsCRP, smoking, alcohol intake, physical activity, and energy intake.	8
Pasdar et al.	2022	Iran	Cross-sectional study	51.25 ± 9.01	N/A	Sarcopenia: Either the low or moderate WC tertile AND the low muscle mass and low muscle strength tertile. Sarcopenic obesity: High tertile of WC AND the low tertile of muscle mass and muscle strength. Hypertension: 140/90 mmHg and/or medication use.	OR (95% CI) for Sarcopenia and hypertension: 0.94 (0.65–1.35); OR (95% CI) for Sarcopenic obesity and hypertension: 1.35 (0.87–2.09).	4,021	Adjusted for age, sex, alcohol, total calorie (kcal), carbohydrate (g), total fat (g), place, education, quantiles of wealth, physical activity.	9
Du et al.	2024	China	Prospective cohort study	66.4 ± 8.5	7 years	Sarcopenia: Based on AWGS 2019 consensus: A decrease in skeletal muscle mass combined with a decrease in physical performance or a decrease in muscle strength. Sarcopenic Obesity: Based on ESPEN and EASO 2022 consensus: The coexistence of obesity and sarcopenia. Hypertension: Assessed based on the questionnaire: “Have you been diagnosed with hypertension by a doctor?”	HR (95% CI) for Sarcopenia and hypertension: 1.17 (0.90–1.52); HR (95% CI) for Sarcopenic obesity and hypertension: 1.61 (1.09–2.37).	7,301	adjusted for sex, age, education, marital, residence, smoke, drink, dyslipidemia, diabetes, kidney disease, eGFR, TG, HDL-C, LDL-C.	7
Kim et al.	2024	Korea	Cross-sectional study	68.9 ± 5.8	N/A	Sarcopenia: DXA-derived ASM/body weight ×100%; moderate sarcopenia = 1 SD below young reference mean, severe sarcopenia = 2 SD below young reference mean; hypertension: SBP ≥ 130 mmHg or DBP ≥ 85 mmHg or antihypertensive medication use	OR (95% CI) for Sarcopenia and hypertension:2.168 (2.020–2.327)	2,237	Adjusted for sex, household income, education, hyperlipidemia medication, smoking, drinking, MVPA, nutrition/total calorie intake, WC, HbA1c, glucose, total cholesterol, HDL-C, triglycerides, AST, ALT	9
Qiu et al.	2024	China	Prospective cohort study	59.7 ± 9.7	9 years	Sarcopenia: AWGS 2019; low muscle mass + low grip strength or low physical performance. Hypertension: self-reported doctor diagnosis or therapy; baseline SBP/DBP > 140/90 mmHg excluded as prevalent hypertension	OR (95% CI) for Sarcopenia and hypertension:0.82 (0.65–1.03)	5,912	Adjusted for age, sex, marital status, residence, education, smoking, drinking, SBP, pulse, BMI, WC, hemoglobin, TG, LDL-C, HDL-C, eGFR, FBG, HbA1c, uric acid, CRP, baseline comorbidities	8
Zou et al.	2025	United Kingdom	Prospective cohort study	Men: 53.01 ± 8.16 Women: 52.55 ± 7.82	5 years	Sarcopenia: EWGSOP2, low grip strength + low SMM/BM; Sarcopenic Obesity: obesity by FM% + sarcopenia; hypertension: ICD-10 I10 new-onset hypertension.	Male: HR(95% CI) for Sarcopenia and hypertension: 3.10 (2.35–4.08); HR(95% CI) for Sarcopenic obesity and hypertension:3.66 (2.98–4.50); Female: HR(95% CI) for Sarcopenia and hypertension: 2.93 (1.95–4.39); HR(95% CI) for Sarcopenic obesity and hypertension:4.04 (3.32–4.91);	Men = 73,152 Women = 109,939	Adjusted for age, education, ethnicity, Townsend deprivation index, smoking, drinking, sleep duration, physical activity, red meat intake, fruit intake, vegetable intake	8

ALM/BMI, appendicular lean mass-to-body mass index ratio; ALT, alanine aminotransferase; ASM/Wt, appendicular skeletal muscle mass-to-body weight ratio; AST, aspartate aminotransferase; AWGS, Asian Working Group for Sarcopenia; BMI, body mass index; BP, blood pressure; CHD, coronary heart disease; CI, confidence interval; CRP, C-reactive protein; DBP, diastolic blood pressure; DXA, dual-energy X-ray absorptiometry; EASO, European Association for the Study of Obesity; eGFR, estimated glomerular filtration rate; ESPEN, European Society for Clinical Nutrition and Metabolism; EWGSOP2, European Working Group on Sarcopenia in Older People 2; FBG, fasting blood glucose; FM%, fat mass percentage; FNIH, Foundation for the National Institutes of Health; HbA1c, glycated hemoglobin A1c; HDL-C, high-density lipoprotein cholesterol; HOMA-IR, homeostatic model assessment of insulin resistance; HR, hazard ratio; hsCRP, high-sensitivity C-reactive protein; ICD-10, international classification of diseases, 10th Revision; LDL-C, low-density lipoprotein cholesterol; MVPA, moderate-to-vigorous physical activity; N/A, not applicable; NOS, Newcastle–Ottawa Scale; OR, odds ratio; SBP, systolic blood pressure; SD, standard deviation; SMM/BM, skeletal muscle mass-to-body mass ratio; TG, triglycerides; WC, waist circumference.

Regarding geographic distribution, four studies were conducted in Korea (26, 30, 32, 33), two studies in China (27, 28), and the remaining three studies were conducted in Lebanon (31), Iran (29), and the United Kingdom (25). In terms of study design, four studies were prospective cohort studies (25, 27, 28, 30), and five studies were cross-sectional studies (26, 29, 31–33). The follow-up duration of the prospective cohort studies ranged from 4 to 9 years.

The diagnostic criteria for sarcopenia varied across studies. Some studies defined sarcopenia according to appendicular skeletal muscle mass adjusted by body weight (26, 30–33), whereas others used criteria based on appendicular lean mass adjusted by BMI, skeletal muscle mass adjusted by body mass, or consensus-based definitions such as the Asian Working Group for Sarcopenia 2019 criteria, the European Working Group on Sarcopenia in Older People 2 criteria, or the Foundation for the National Institutes of Health Sarcopenia Project criteria (25, 27–29). Sarcopenic obesity was generally defined as the coexistence of sarcopenia and obesity, but obesity indicators differed across studies and included waist circumference, BMI, body fat percentage, fat mass percentage, or other body-composition-based measures.

Hypertension definitions also varied among the included studies. Most studies defined hypertension according to measured blood pressure thresholds, self-reported physician diagnosis, medical history, or current use of antihypertensive medication, while one study used ICD-10 code I10 to identify incident hypertension (25). Among the 9 included studies, 7 studies reported ORs, and 2 studies reported HRs. Effect estimates were adjusted for different sets of covariates, most commonly including age, sex, lifestyle factors, anthropometric indices, metabolic variables, and comorbidities. The detailed characteristics of the included studies are shown in Table 3.

### 3.3 Quality assessment

According to the study-design-specific NOS assessments, the included studies were generally of moderate-to-high methodological quality. Among the four prospective cohort studies, all were rated as high quality. Among the five cross-sectional studies, four were rated as high quality and one was rated as moderate quality. The detailed item-level quality assessment results are shown in Tables 1, 2.

### 3.4 Meta-analysis

#### 3.4.1 Association between sarcopenia and hypertension

Eight studies, comprising 10 independent datasets and 346,564 participants, were included in the quantitative analysis of the association between sarcopenia and hypertension. Substantial heterogeneity was observed across the included datasets (I^2^ = 95.51%, τ^2^ = 0.20, *p* < 0.001). Therefore, a random-effects model using the restricted maximum likelihood (REML) method was applied.

**Figure 2:**
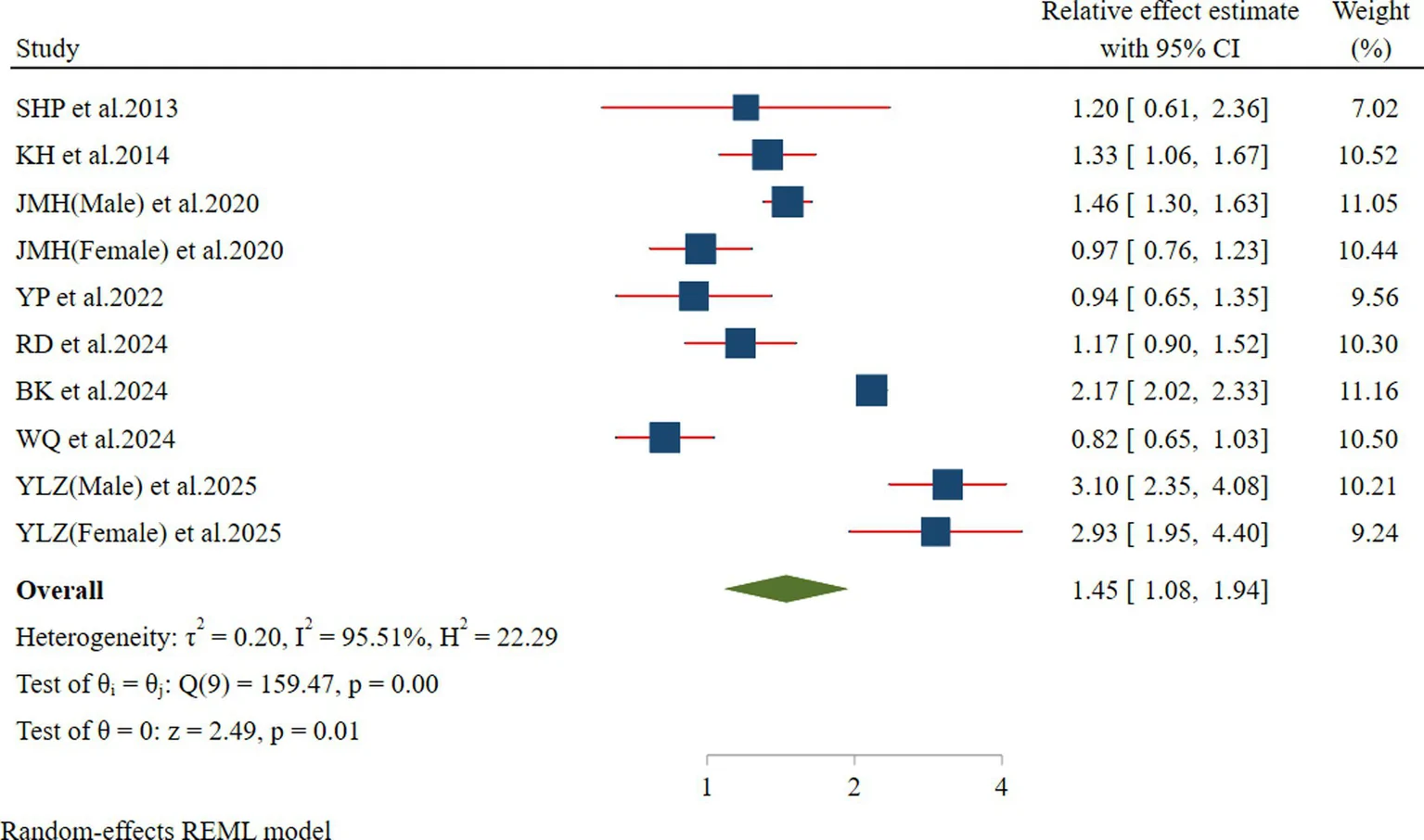
Forest plot of the association between sarcopenia and hypertension.

As shown in Figure 2, the pooled analysis demonstrated a statistically significant positive association between sarcopenia and hypertension, with a pooled relative effect estimate of 1.45 (95% CI: 1.08–1.94; *p* = 0.01). The individual effect estimates varied across datasets, and five of the 10 datasets had 95% CIs crossing the null value of 1.0. Nevertheless, the overall pooled estimate remained statistically significant.

**Figure 3:**
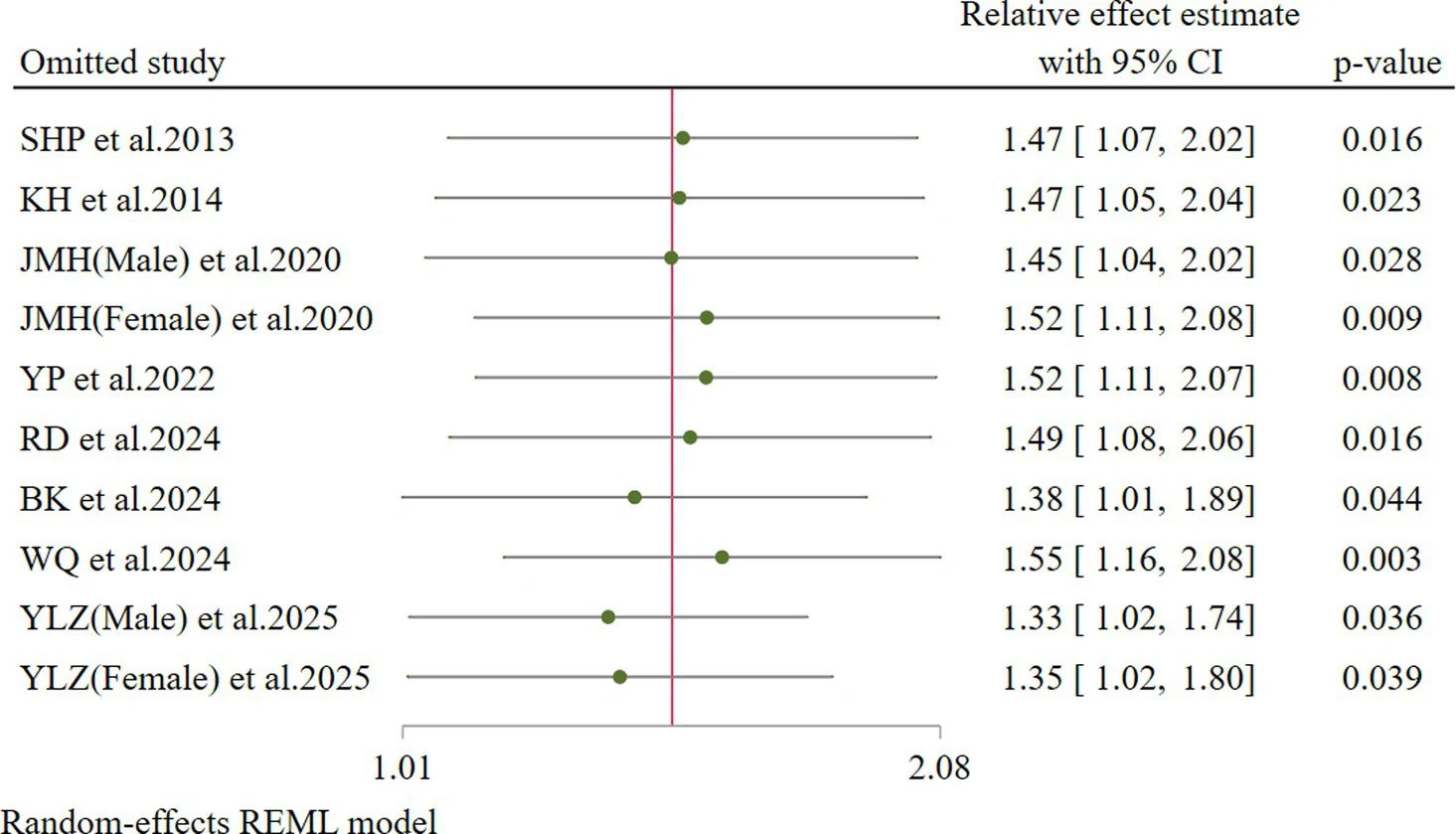
Sensitivity analysis of the association between sarcopenia and hypertension.

A leave-one-out sensitivity analysis was subsequently performed to examine the influence of individual datasets on the pooled estimate. As shown in Figure 3, sequential exclusion of each dataset yielded pooled effect estimates ranging from 1.33 to 1.55. All recalculated 95% CIs remained above the null value of 1.0, and the corresponding *p* values ranged from 0.003 to 0.044. Thus, the direction and statistical significance of the pooled association were retained after the exclusion of any single dataset.

**Figure 4:**
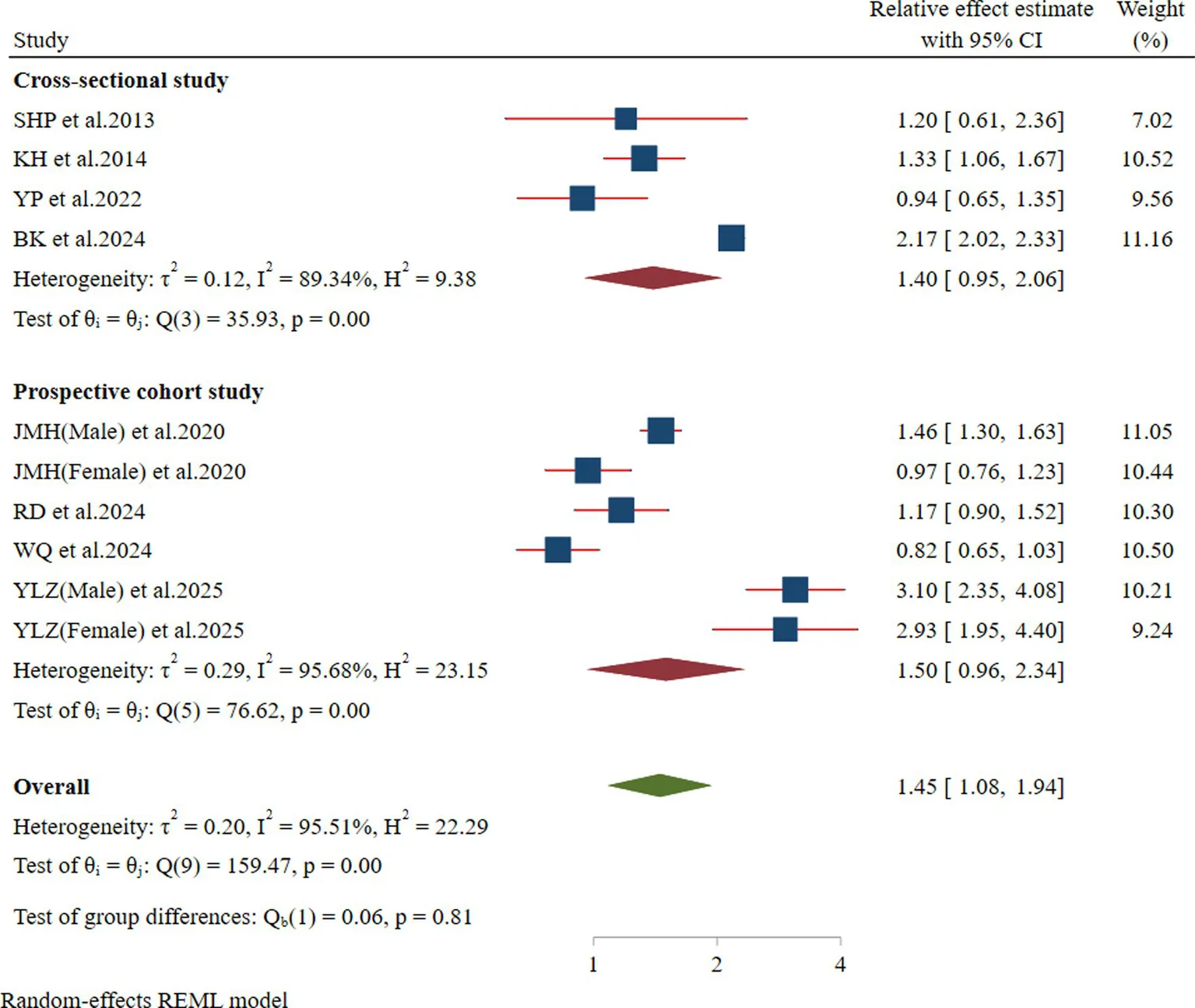
Subgroup analysis by study design for the association between sarcopenia and hypertension.

An additional design-restricted sensitivity analysis was conducted by limiting the analysis to prospective cohort studies. Four prospective cohort studies, comprising six independent datasets, were included in this analysis. The pooled relative effect estimate was 1.50 (95% CI: 0.96–2.34). Although the direction of the association remained positive, the 95% CI included the null value of 1.0 and the pooled association did not reach statistical significance. The corresponding estimate is also presented in the study-design subgroup analysis in Figure 4.

#### 3.4.2 Association between sarcopenic obesity and hypertension

Six studies, comprising seven independent datasets and 206,275 participants, were included in the quantitative analysis of the association between sarcopenic obesity and hypertension. Substantial heterogeneity was observed across the included datasets (I^2^ = 86.85%, τ^2^ = 0.18, *p* < 0.001). Therefore, a random-effects model using the restricted maximum likelihood (REML) method was applied.

**Figure 5:**
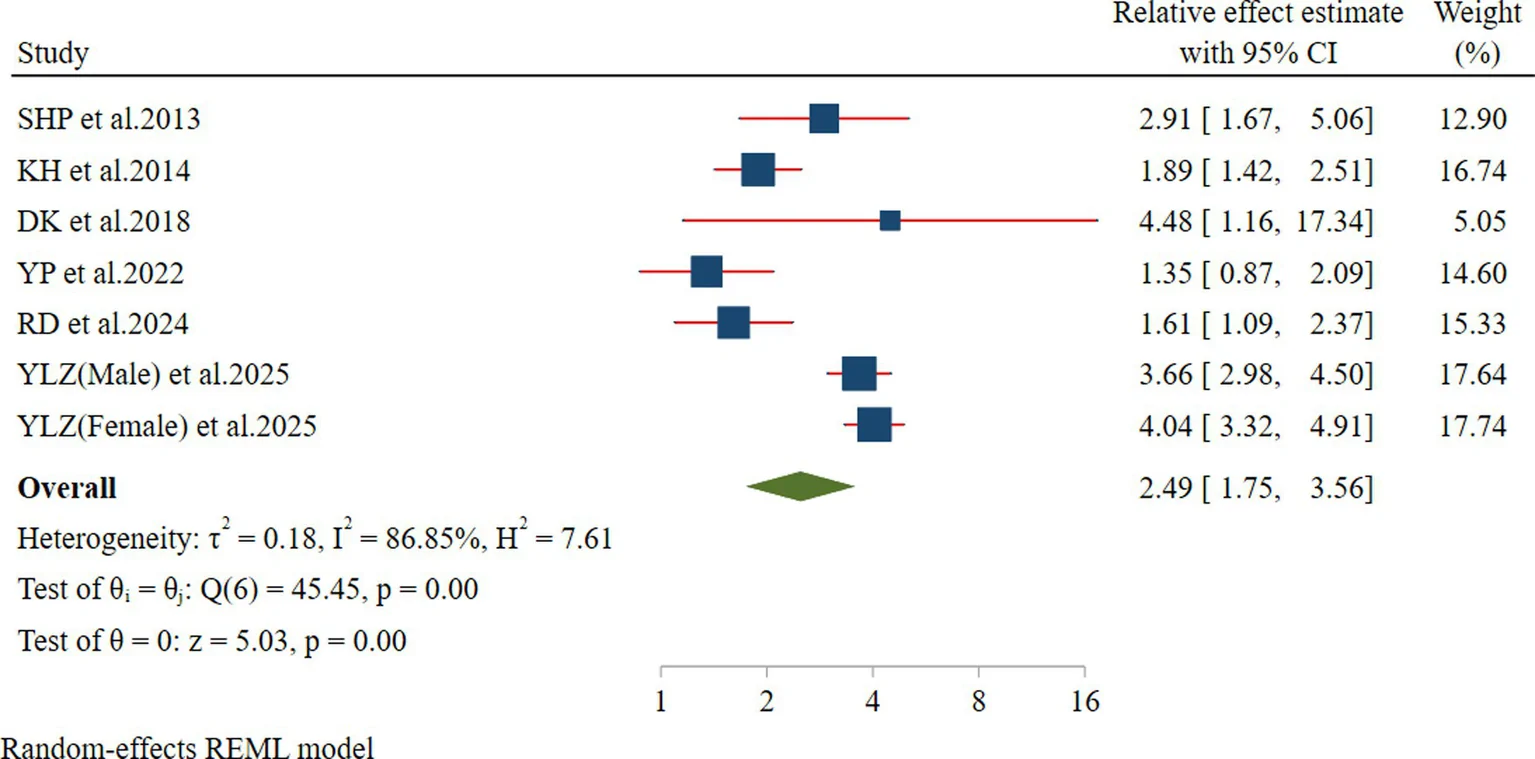
Forest plot of the association between sarcopenic obesity and hypertension.

As shown in Figure 5, the pooled analysis demonstrated a statistically significant positive association between sarcopenic obesity and hypertension, with a pooled relative effect estimate of 2.49 (95% CI: 1.75–3.56; *p* < 0.001). Individual effect estimates varied across the included datasets. The 95% CI of the Pasdar et al. (29) dataset crossed the null value of 1.0, whereas the remaining datasets showed statistically significant positive associations. The overall pooled estimate remained statistically significant.

**Figure 6:**
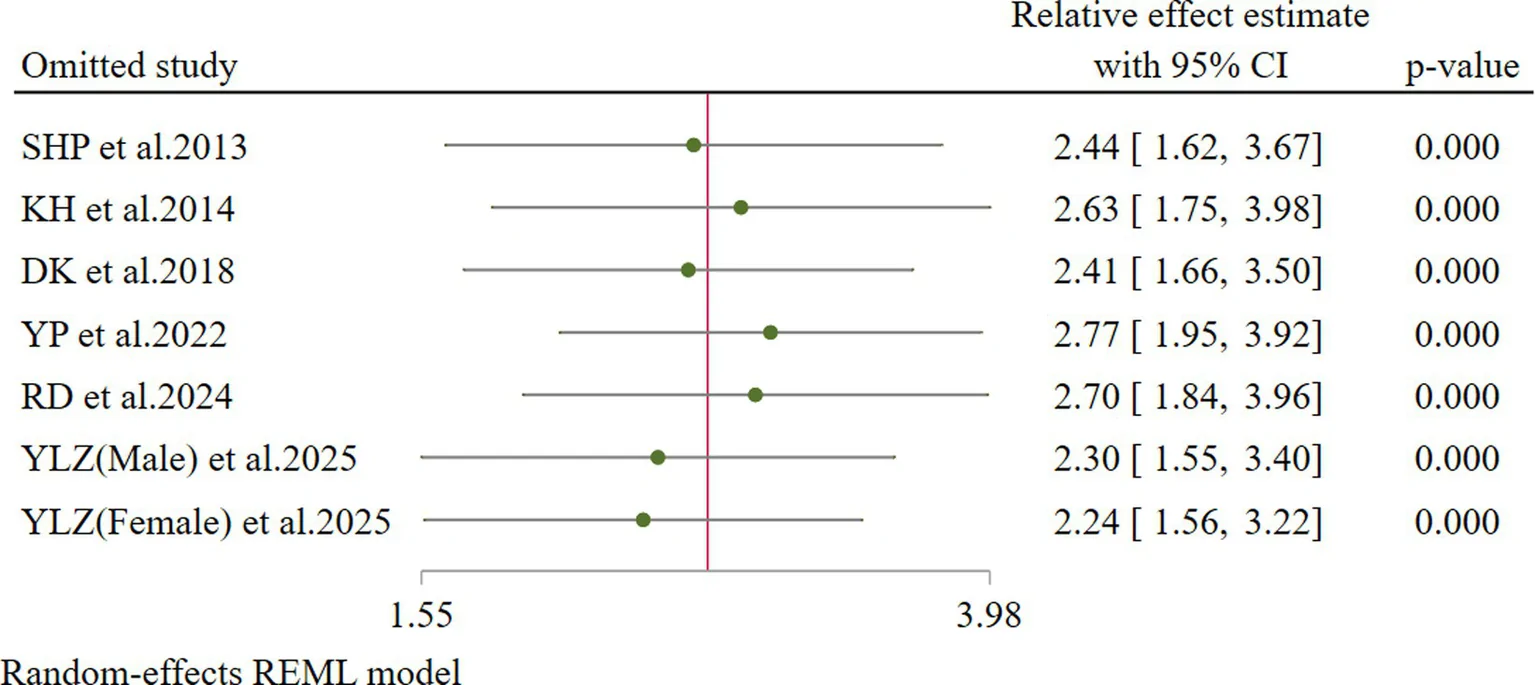
Sensitivity analysis of the association between sarcopenic obesity and hypertension.

A leave-one-out sensitivity analysis was subsequently performed to examine the influence of individual datasets on the pooled estimate. As shown in Figure 6, sequential exclusion of each dataset yielded pooled effect estimates ranging from 2.24 to 2.77. All recalculated 95% CIs remained above the null value of 1.0, and the corresponding *p* values were < 0.001. Thus, the direction and statistical significance of the pooled association were retained after the exclusion of any single dataset.

**Figure 7:**
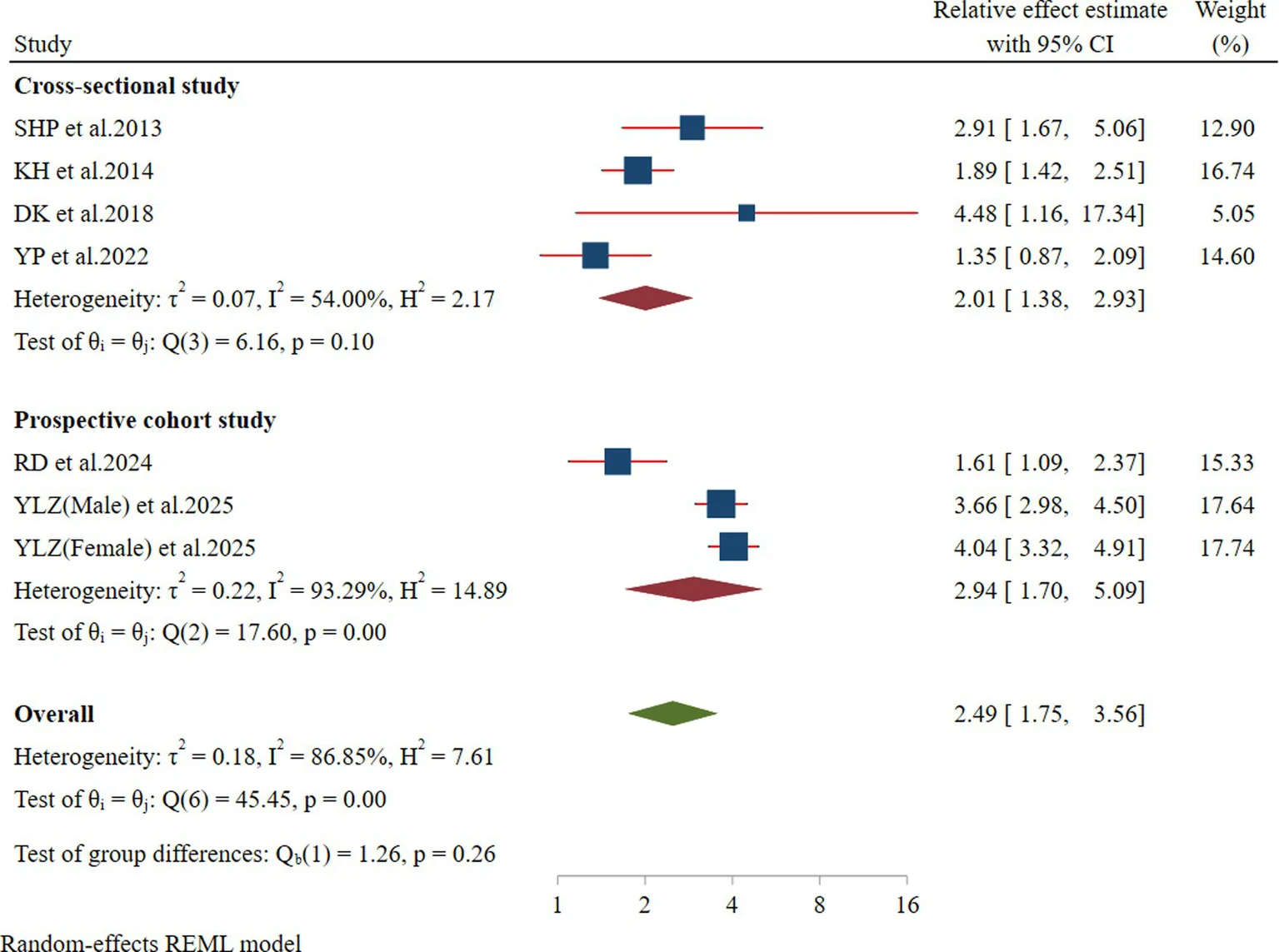
Subgroup analysis by study design for the association between sarcopenic obesity and hypertension.

An additional design-restricted sensitivity analysis was performed by restricting the analysis to prospective cohort studies. Two prospective cohort studies, comprising three independent datasets, were included. The pooled relative effect estimate was 2.94 (95% CI: 1.70–5.09), and the positive association remained statistically significant in the cohort-restricted analysis. The corresponding estimate is also presented in the study-design subgroup analysis in Figure 7.

#### 3.4.3 Subgroup analysis

##### 3.4.3.1 Subgroup analysis of sarcopenia and hypertension

###### 3.4.3.1.1 Subgroup analysis by study design

Subgroup analysis according to study design is presented in Figure 4. Consistent with the design-restricted sensitivity analysis reported above, the pooled relative effect estimate was 1.50 (95% CI: 0.96–2.34) for prospective cohort studies, compared with 1.40 (95% CI: 0.95–2.06) for cross-sectional studies. Neither subgroup estimate reached statistical significance, and no significant difference was observed between the two study-design subgroups (*p* = 0.81 for subgroup differences).

###### 3.4.3.1.2 Subgroup analysis by age

**Figure 8:**
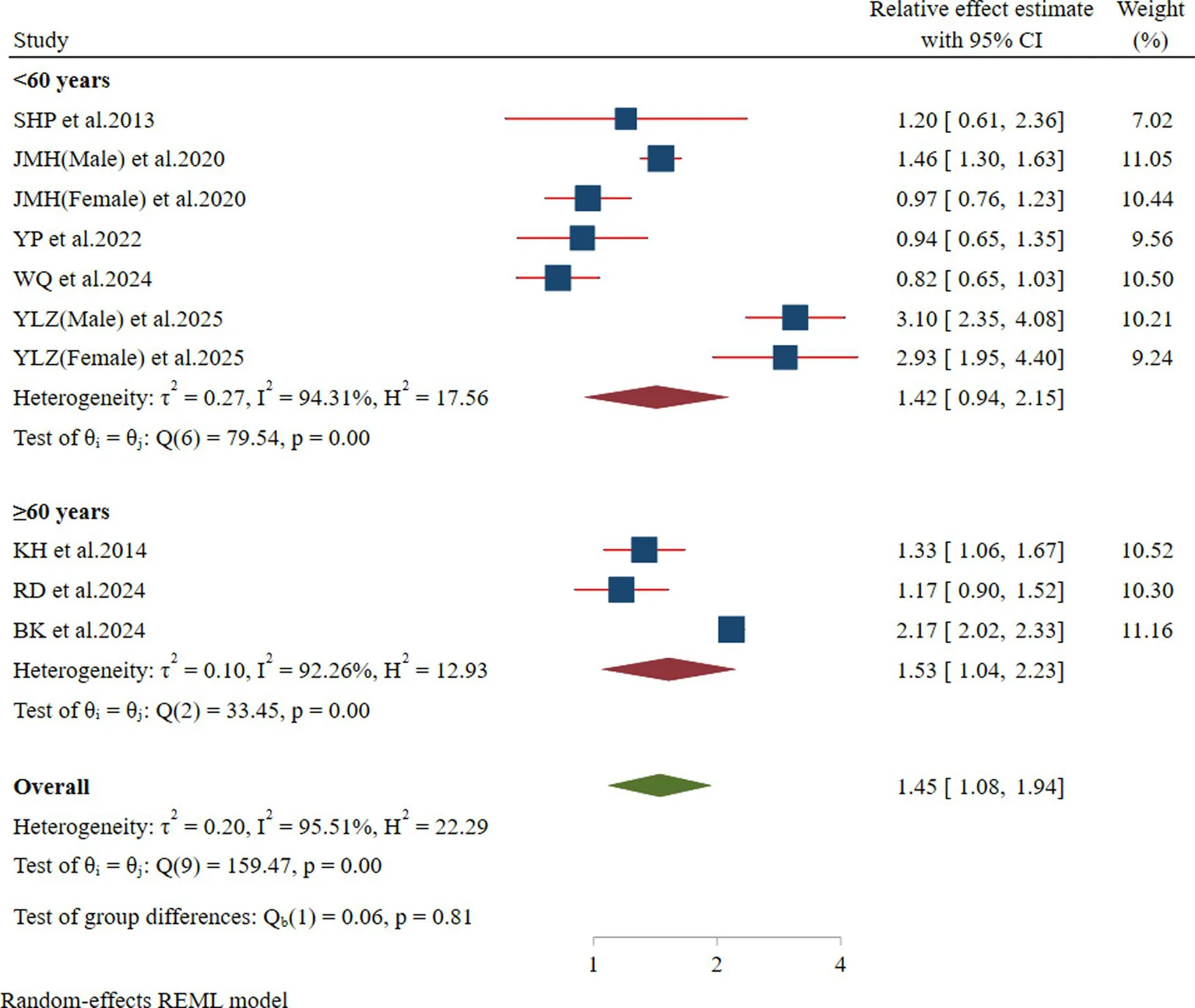
Subgroup analysis by age for the association between sarcopenia and hypertension.

As shown in Figure 8, the pooled relative effect estimate was 1.42 (95% CI: 0.94–2.14) in the <60 years subgroup and 1.53 (95% CI: 1.04–2.23) in the ≥60 years subgroup. The association was statistically significant in the ≥60 years subgroup but not in the <60 years subgroup. However, the test for subgroup differences was not statistically significant (*p* = 0.81).

###### 3.4.3.1.3 Subgroup analysis by sarcopenia diagnostic criteria

**Figure 9:**
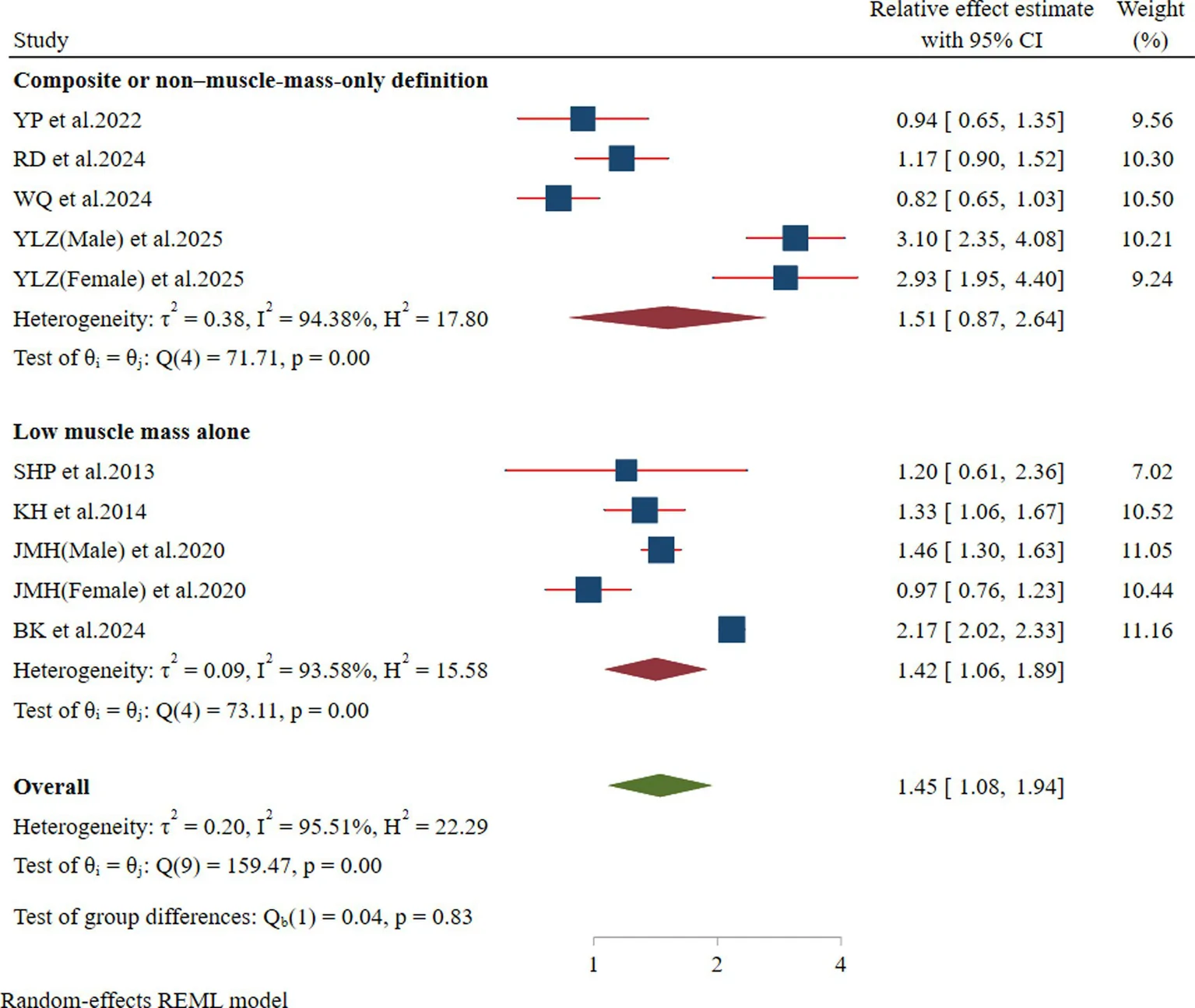
Subgroup analysis by sarcopenia diagnostic criteria for the association between sarcopenia and hypertension.

Subgroup analysis according to sarcopenia diagnostic criteria is shown in Figure 9. Studies using composite or non-muscle-mass-only definitions yielded a pooled relative effect estimate of 1.51 (95% CI: 0.87–2.64), whereas studies defining sarcopenia on the basis of low muscle mass alone yielded an estimate of 1.42 (95% CI: 1.06–1.89). The test for subgroup differences showed no statistically significant difference between the two categories (*p* = 0.83).

###### 3.4.3.1.4 Subgroup analysis by hypertension definition

**Figure 10:**
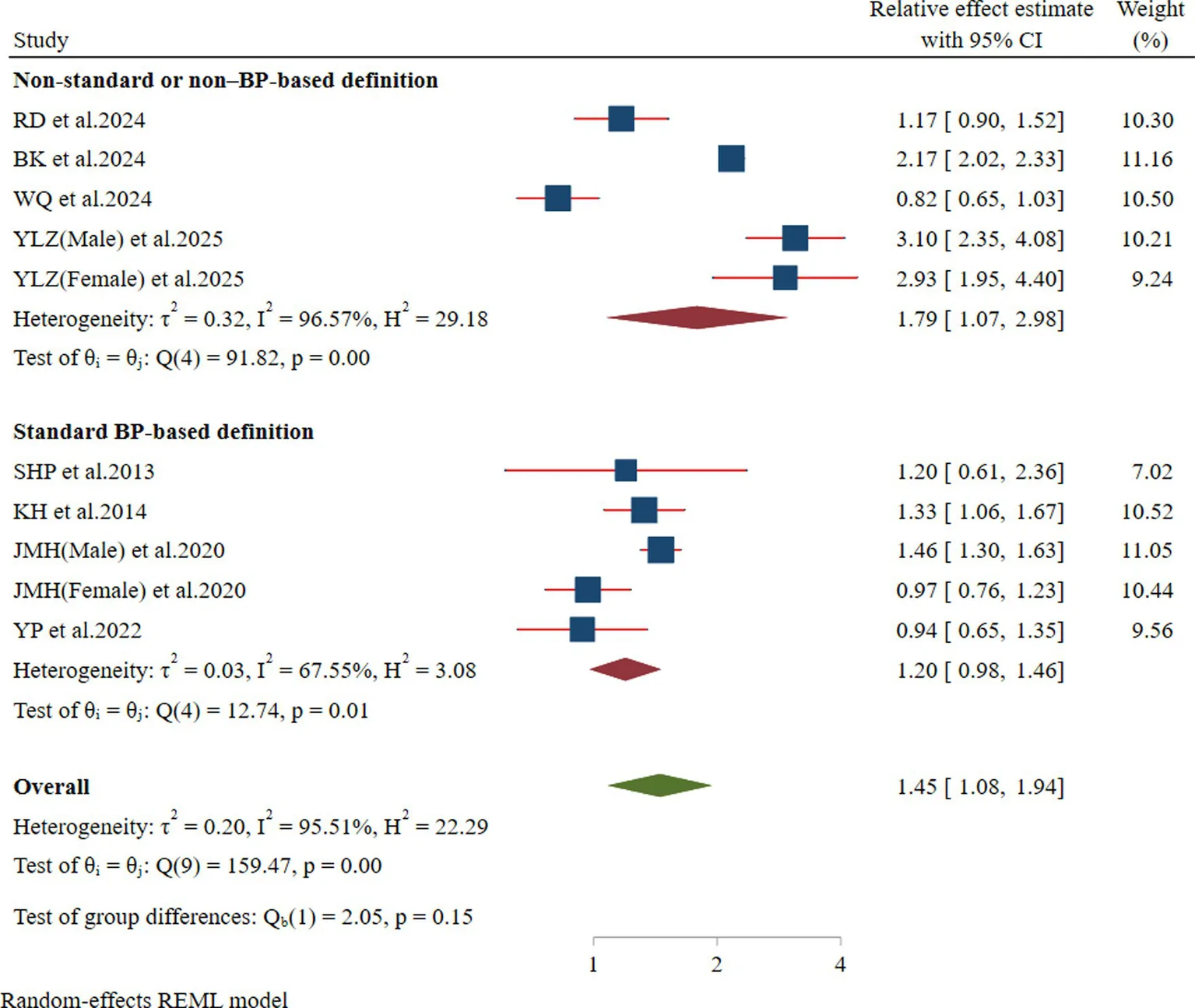
Subgroup analysis by hypertension definition for the association between sarcopenia and hypertension.

As presented in Figure 10, studies using non-standard or non-BP-based hypertension definitions yielded a pooled relative effect estimate of 1.79 (95% CI: 1.07–2.98), whereas studies using standard BP-based definitions yielded an estimate of 1.20 (95% CI: 0.98–1.46). The difference between the two subgroups was not statistically significant (*p* = 0.15 for subgroup differences).

###### 3.4.3.1.5 Exploratory analysis by effect measure type

As shown in Supplementary Figure S1, the pooled relative effect estimate was 2.18 (95% CI: 1.16–4.09) among studies reporting HRs (two studies, three independent datasets) and 1.23 (95% CI: 0.94–1.62) among studies reporting ORs (six studies, seven independent datasets). Substantial heterogeneity remained within both strata (HR: I^2^ = 92.04%; OR: I^2^ = 93.68%). The test for subgroup differences was not statistically significant (*p* = 0.10).

##### 3.4.3.2 Subgroup analysis of sarcopenic obesity and hypertension

###### 3.4.3.2.1 Subgroup analysis by study design

Subgroup analysis according to study design is presented in Figure 7. Consistent with the cohort-restricted sensitivity analysis, the pooled relative effect estimate was 2.94 (95% CI: 1.70–5.09) for prospective cohort studies and 2.01 (95% CI: 1.38–2.93) for cross-sectional studies. Statistically significant positive associations were observed in both subgroups, with no statistically significant difference between them (*p* = 0.26 for subgroup differences).

###### 3.4.3.2.2 Subgroup analysis by age

**Figure 11:**
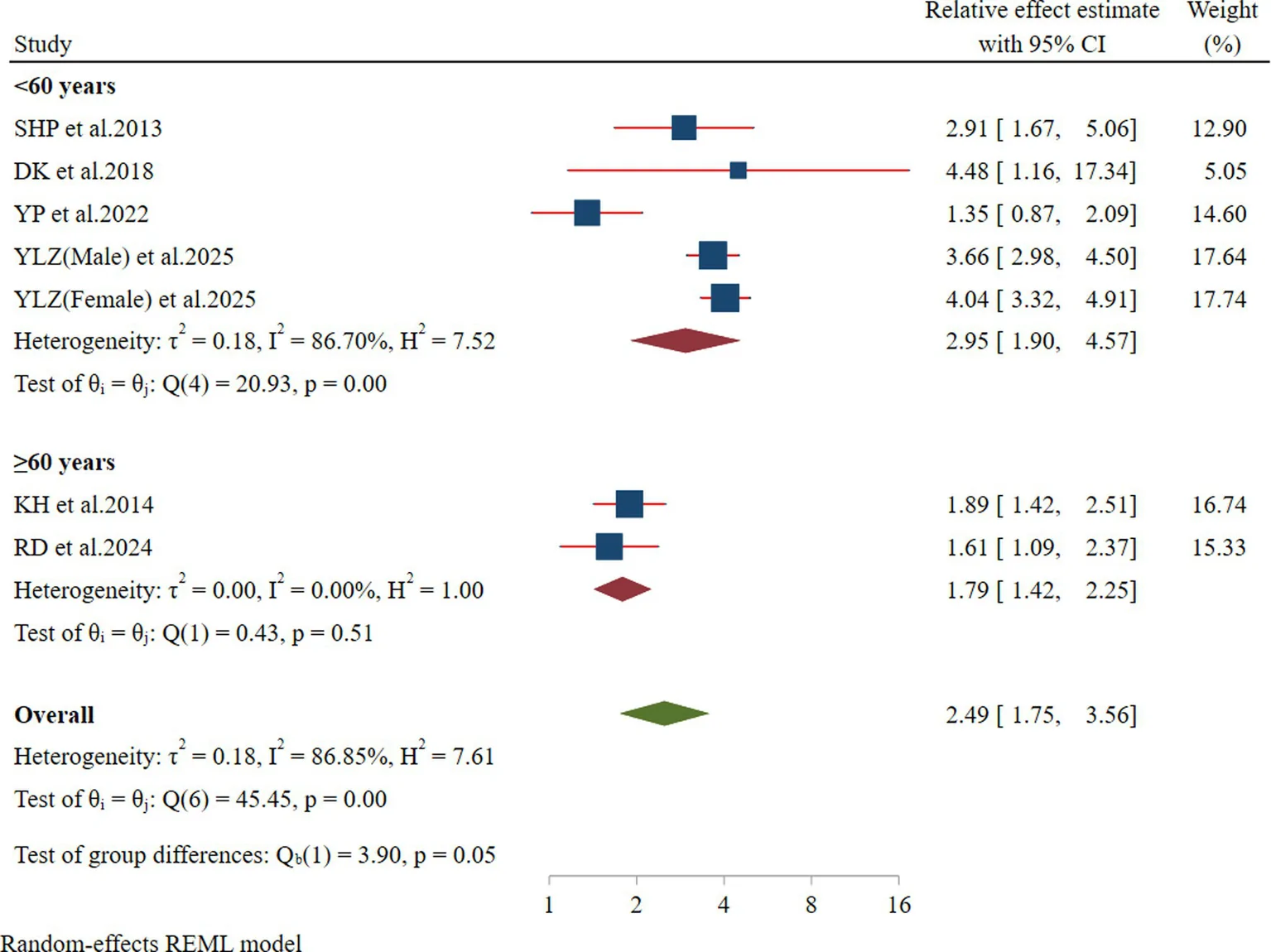
Subgroup analysis by age for the association between sarcopenic obesity and hypertension.

As shown in Figure 11, the pooled relative effect estimate was 2.95 (95% CI: 1.90–4.57) in the <60 years subgroup and 1.79 (95% CI: 1.42–2.25) in the ≥60 years subgroup. Both subgroup estimates were statistically significant. The test for subgroup differences yielded a *p* value of 0.05.

###### 3.4.3.2.3 Subgroup analysis by sarcopenia diagnostic criteria

**Figure 12:**
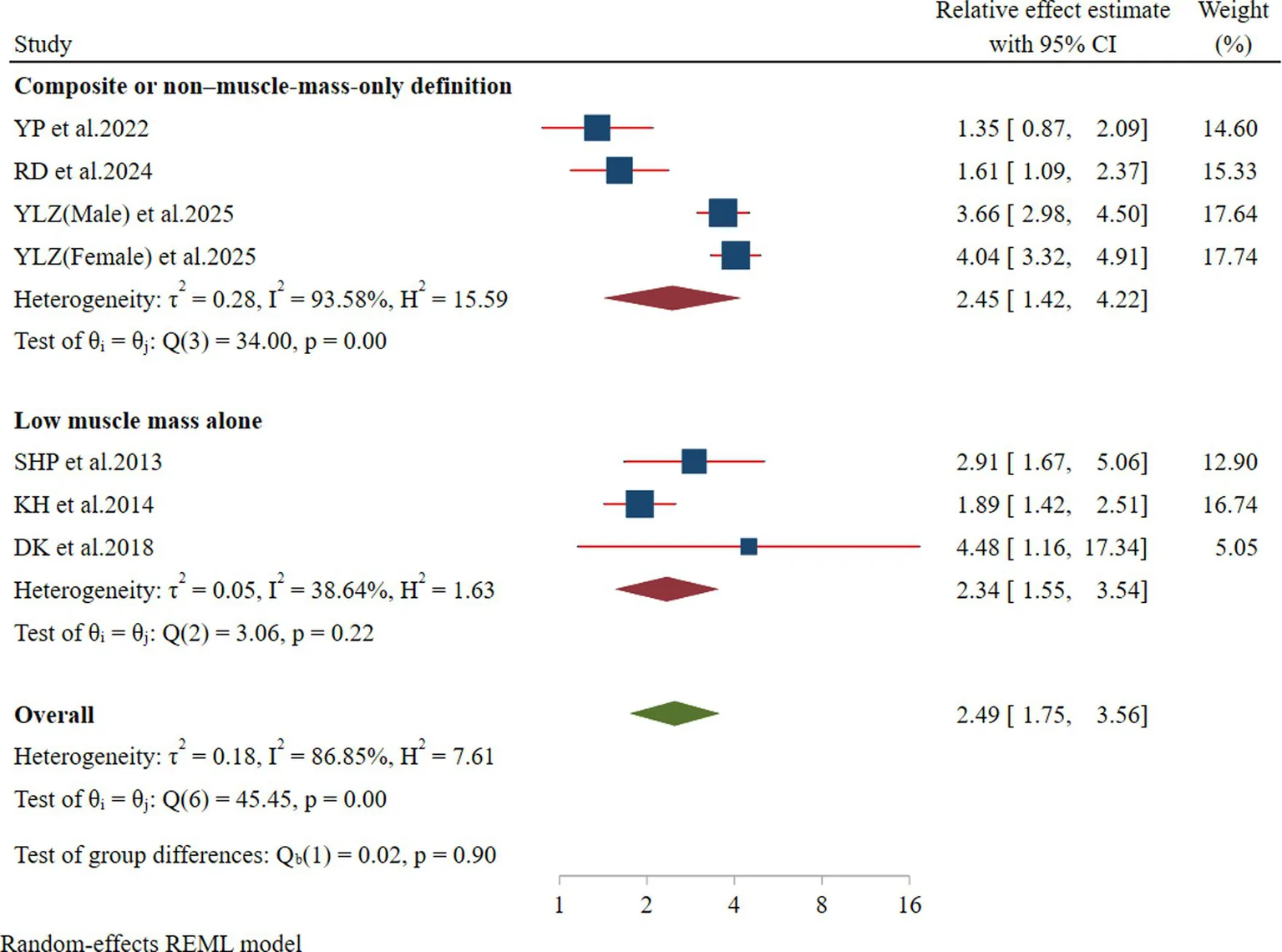
Subgroup analysis by sarcopenia diagnostic criteria for the association between sarcopenic obesity and hypertension.

Subgroup analysis according to sarcopenia diagnostic criteria is presented in Figure 12. The pooled relative effect estimate was 2.45 (95% CI: 1.42–4.22) for studies using composite or non-muscle-mass-only definitions and 2.34 (95% CI: 1.55–3.54) for studies defining sarcopenia on the basis of low muscle mass alone. Both subgroup estimates were statistically significant, and no significant difference was observed between the two categories (*p* = 0.90 for subgroup differences).

###### 3.4.3.2.4 Subgroup analysis by hypertension definition

**Figure 13:**
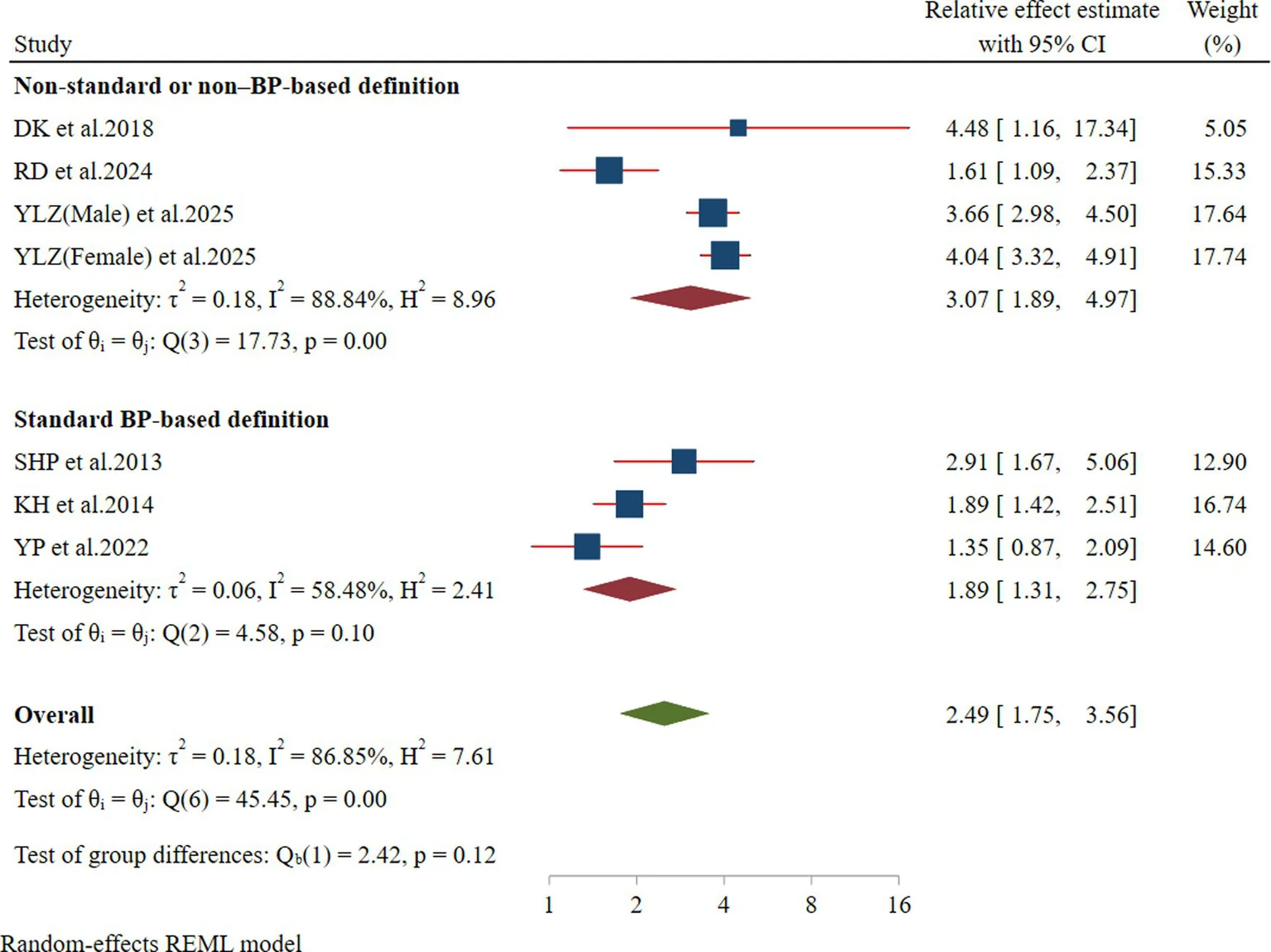
Subgroup analysis by hypertension definition for the association between sarcopenic obesity and hypertension.

As shown in Figure 13, the pooled relative effect estimate was 3.07 (95% CI: 1.89–4.97) among studies using non-standard or non-BP-based hypertension definitions and 1.89 (95% CI: 1.31–2.75) among studies using standard BP-based definitions. Statistically significant associations were observed in both subgroups, whereas the test for subgroup differences was not statistically significant (*p* = 0.12).

###### 3.4.3.2.5 Exploratory analysis by effect measure type

As shown in Supplementary Figure S2, the pooled relative effect estimate was 2.94 (95% CI: 1.70–5.09) among studies reporting HRs (two studies, three independent datasets) and 2.01 (95% CI: 1.38–2.93) among studies reporting ORs (four studies, four independent datasets). Statistically significant positive associations were observed in both strata. Heterogeneity was substantial in the HR stratum (I^2^ = 93.29%) and moderate in the OR stratum (I^2^ = 54.00%). The test for subgroup differences was not statistically significant (*p* = 0.26).

#### 3.4.4 Publication bias

**Figure 14:**
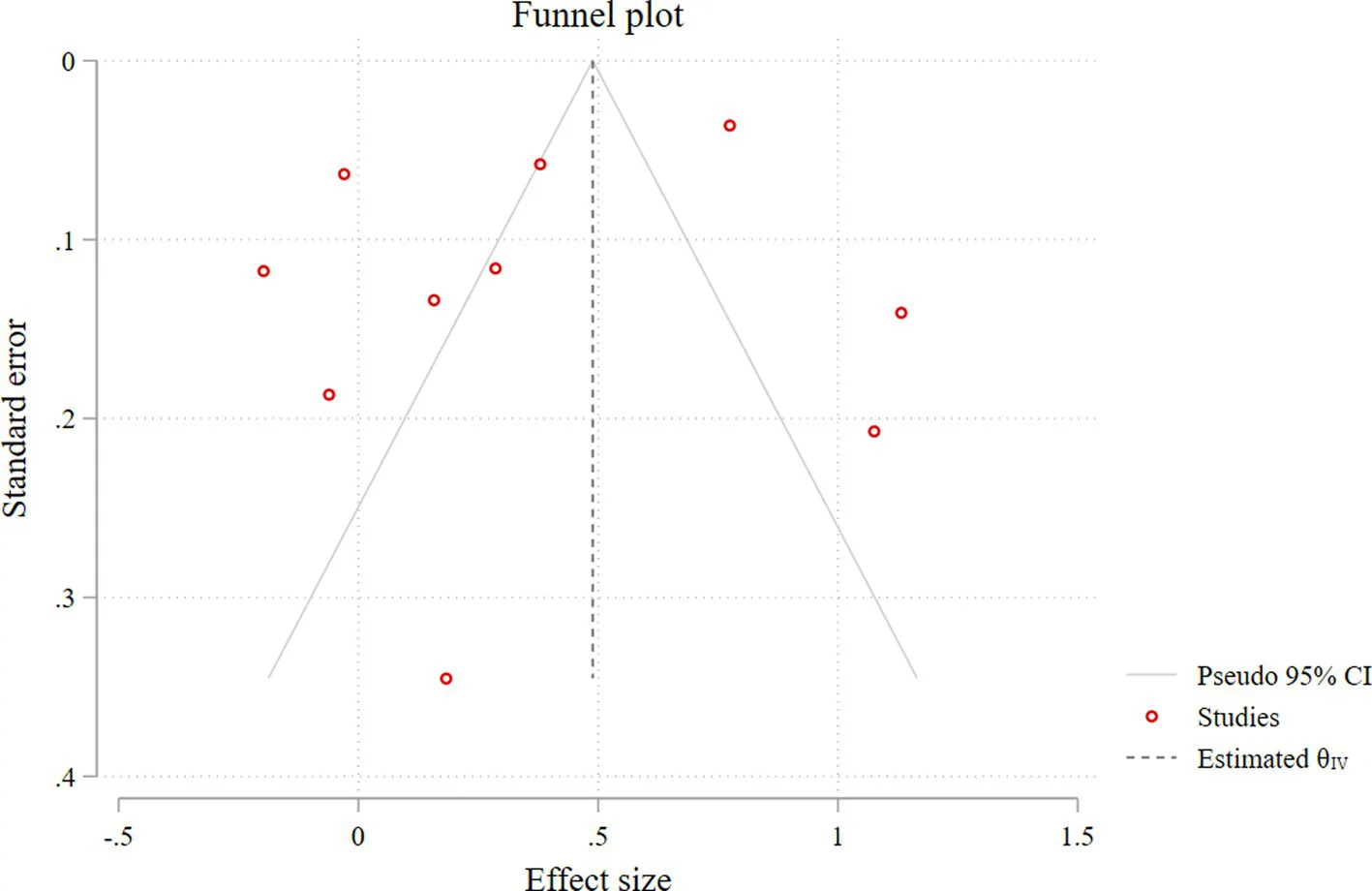
Funnel plot for the association between sarcopenia and hypertension.

**Figure 15:**
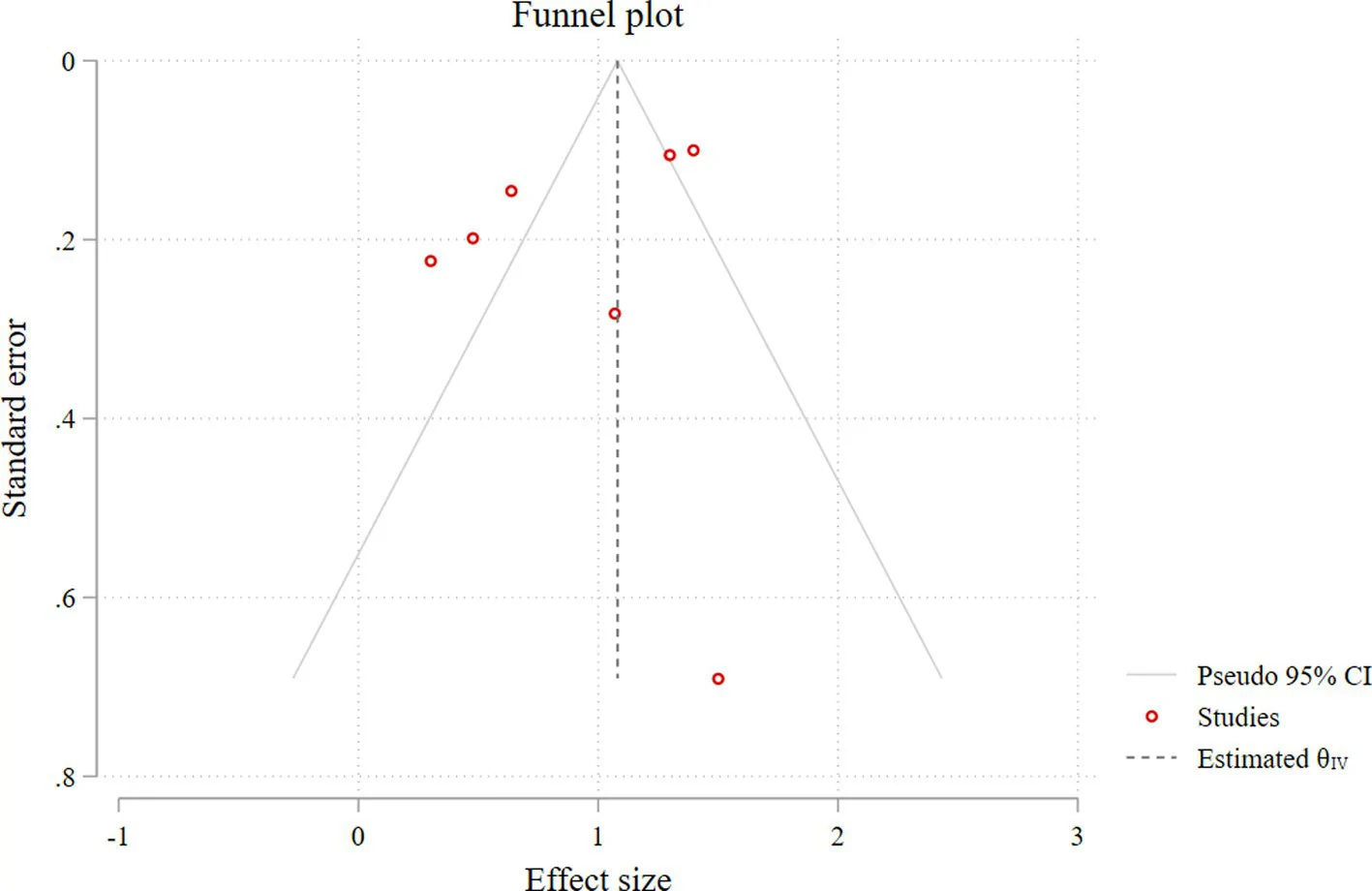
Funnel plot for the association between sarcopenic obesity and hypertension.

Visual inspection of the funnel plots for the associations of sarcopenia and sarcopenic obesity with hypertension did not reveal marked asymmetry (Figures 14, 15). Exploratory Egger’s regression tests yielded *p* values of 0.9949 for the sarcopenia analysis and 0.9049 for the sarcopenic obesity analysis. However, because fewer than 10 studies were included in each meta-analysis, the statistical power of funnel plot asymmetry tests was limited. Therefore, these findings should be interpreted cautiously and cannot exclude the possibility of publication bias or other small-study effects.

## 4 Discussion

### 4.1 Main findings

This systematic review and meta-analysis synthesized observational evidence from nine studies examining the associations of sarcopenia and sarcopenic obesity with hypertension. Sarcopenia was significantly associated with hypertension, with a pooled relative effect estimate of 1.45 (95% CI: 1.08–1.94), while sarcopenic obesity was also significantly associated with hypertension, with a pooled estimate of 2.49 (95% CI: 1.75–3.56). Although the pooled estimate was numerically larger for sarcopenic obesity, the two exposure-specific estimates were derived from overlapping but non-identical sets of studies and were not formally compared. Therefore, the difference in magnitude should not be interpreted as evidence of statistical interaction or synergy.

Substantial between-study heterogeneity was observed in both primary analyses. Subgroup analyses according to study design, age, sarcopenia diagnostic criteria, and hypertension definition generally showed associations in the same direction, although most tests for subgroup differences were not statistically significant. Leave-one-out analyses retained the direction and statistical significance of both primary pooled associations. In analyses restricted to prospective cohort studies, the association between sarcopenia and hypertension remained positive but did not reach statistical significance, whereas the association between sarcopenic obesity and hypertension remained statistically significant. Exploratory analyses stratified by effect measure type did not identify statistically significant differences between OR- and HR-based estimates for sarcopenia (*p* = 0.10) or sarcopenic obesity (*p* = 0.26); however, only two studies contributed HR estimates for each exposure. Overall, the findings support associations of both sarcopenia and sarcopenic obesity with hypertension, while uncertainty remains regarding the magnitude, consistency, and temporal nature of these associations.

### 4.2 Interpretation of findings

The present study extends previous evidence by evaluating sarcopenia and sarcopenic obesity within the same systematic framework and across a broader adult age range. Bai et al. previously synthesized evidence on the association between sarcopenia and hypertension specifically in older adults, whereas the present review separately evaluated sarcopenia and sarcopenic obesity and incorporated additional evidence from younger and middle-aged adults (16). Differences between the present and previous pooled estimates may reflect variation in age distribution, exposure definitions, study design, hypertension ascertainment, effect measures, and the evidence available at the time of each review. The larger pooled estimate observed for sarcopenic obesity should be regarded as a descriptive difference rather than evidence that obesity statistically modifies or amplifies the association between sarcopenia and hypertension, because formal interaction analyses were not available.

The observed associations are biologically plausible, although the present meta-analysis cannot establish the mechanisms underlying them. Skeletal muscle is a major site of insulin-mediated glucose disposal and contributes to metabolic and vascular regulation through endocrine signaling. Reduced muscle quantity or impaired muscle function may be accompanied by lower glucose utilization, altered myokine signaling, reduced physical activity, and worsening insulin sensitivity. Excess adiposity, particularly visceral adiposity, is associated with chronic low-grade inflammation, oxidative stress, insulin resistance, endothelial dysfunction, and dysregulation of neurohormonal pathways involved in blood pressure regulation. The coexistence of impaired muscle health and excess adiposity may therefore identify a metabolically adverse phenotype in which several hypertension-related pathways coexist. These mechanisms provide biological plausibility for the epidemiological associations observed in the present study but were not directly tested in the included studies and should not be interpreted as evidence of a specific causal pathway.

The age-stratified findings require cautious interpretation. For sarcopenia, a statistically significant association was observed in the ≥60-year subgroup but not in the <60-year subgroup; however, the test for subgroup differences was not statistically significant. Thus, the available evidence does not demonstrate that age modifies the association between sarcopenia and hypertension. For sarcopenic obesity, statistically significant associations were observed in both age groups, with a numerically larger estimate among participants aged <60 years and a borderline subgroup-difference *p* value of 0.05. These findings suggest that the association between sarcopenic obesity and hypertension is not confined to older adults, but they do not establish a stronger association in younger populations. Differences in the underlying phenotype across the life course may partly contribute to the observed pattern: muscle impairment in younger and middle-aged adults may more often coexist with obesity, physical inactivity, nutritional imbalance, and metabolic dysfunction, whereas age-related neuromuscular and body-composition changes may play a greater role in older adults. Given the limited number of datasets within each age stratum, these interpretations remain exploratory.

The design-restricted analyses provide additional context for interpreting the overall findings. When the analysis was limited to prospective cohort studies, the pooled estimate for sarcopenia remained positive but was imprecise and included the null value, whereas the association between sarcopenic obesity and hypertension remained statistically significant. These findings indicate that the overall positive direction of the associations was not solely attributable to cross-sectional evidence, particularly for sarcopenic obesity. Nevertheless, the number of prospective studies was small and the corresponding confidence intervals were relatively wide. Although prospective cohort studies improve temporal ordering compared with cross-sectional studies, they do not eliminate residual confounding or establish causality. The longitudinal findings should therefore be regarded as supportive but still inconclusive.

Exploratory analyses stratified by effect measure type provide additional methodological context. For sarcopenia, studies reporting HRs yielded a numerically larger pooled estimate than studies reporting ORs, whereas for sarcopenic obesity both effect-measure strata showed statistically significant positive associations. No statistically significant subgroup difference was detected for either exposure. However, substantial heterogeneity persisted within the effect-measure strata, and only two studies contributed HR estimates for each exposure. Moreover, effect measure type partly overlapped with study design and outcome timing, particularly in the sarcopenic obesity analysis. Therefore, these stratified analyses should be viewed as exploratory stratified analyses rather than evidence that ORs and HRs are interchangeable or that the observed differences are attributable to effect measure type itself.

Interpretation of the pooled estimates is further complicated by heterogeneity in exposure and outcome definitions. Sarcopenia was operationalized using different combinations of muscle mass, muscle strength, and physical performance. Some studies relied predominantly on low muscle mass, whereas others applied multidimensional consensus-based criteria. In individuals with obesity, muscle mass indices normalized to height alone may inadequately capture relative muscle deficiency because they do not account for the influence of larger body size. The ESPEN/EASO consensus therefore recommends assessing relative muscle mass using body-weight-adjusted indices, whereas the Japanese Working Group on Sarcopenic Obesity adopts BMI-adjusted limb skeletal muscle mass in its diagnostic framework (34, 35). Expert commentary has further highlighted the ongoing methodological debate regarding whether body weight alone is sufficient to represent body size when defining the sarcopenia component of sarcopenic obesity (36). Moreover, studies evaluating sarcopenia did not always clearly distinguish participants with concomitant obesity, raising the possibility that some sarcopenia groups included heterogeneous body-composition phenotypes. Definitions of sarcopenic obesity also differed because obesity was assessed using BMI, waist circumference, body fat percentage, or other body-composition measures, with thresholds varying by population and sex. Related but distinct phenotypes, such as osteosarcopenic obesity, should also be clearly distinguished from sarcopenic obesity because the additional presence of impaired bone health may identify a clinically different population (37).

Hypertension ascertainment was likewise heterogeneous, ranging from measured blood pressure thresholds to physician diagnosis, medication use, medical history, or diagnostic codes. Although subgroup analyses did not demonstrate statistically significant differences according to sarcopenia diagnostic criteria or hypertension definition, this should not be interpreted as evidence that the definitions are interchangeable. The limited number of studies within individual strata reduced the statistical power to detect genuine subgroup differences.

The substantial heterogeneity observed in both primary analyses is central to the interpretation of the findings. The pooled estimates should be understood as average associations across clinically and methodologically diverse studies rather than as uniform effects applicable to all populations and settings. The absence of statistically significant subgroup differences for most prespecified factors does not exclude their contribution to heterogeneity. Differences in age distribution, sex composition, comorbidity burden, socioeconomic characteristics, lifestyle patterns, body-composition assessment, exposure thresholds, hypertension ascertainment, follow-up duration, and covariate adjustment may all have contributed to between-study variability. Larger prospective studies using harmonized exposure and outcome definitions, as well as individual participant data meta-analyses where feasible, would help clarify whether these characteristics meaningfully modify the observed associations.

### 4.3 Implications and limitations

From a clinical and public health perspective, the findings suggest that muscle health and adiposity may provide complementary information when characterizing cardiometabolic health. The associations observed across different adult age groups also indicate that adverse muscle–adiposity phenotypes warrant investigation beyond exclusively geriatric populations. Future risk-stratification research should evaluate whether multidimensional assessment incorporating muscle mass, muscle strength or physical performance, adiposity, and established cardiovascular risk factors improves hypertension prediction beyond conventional measures. However, the present meta-analysis did not evaluate the predictive performance, cost-effectiveness, or clinical outcomes of such screening strategies, nor did it establish that treating sarcopenia or sarcopenic obesity prevents hypertension. The findings therefore support further prospective and interventional research rather than routine screening or specific clinical recommendations.

Several limitations should be considered. First, the evidence base consisted entirely of observational studies, including a substantial proportion of cross-sectional analyses. Consequently, the overall meta-analysis cannot establish temporality or causality. The prospective cohort analyses provided useful longitudinal information, but the number of such studies was limited, and the cohort-restricted estimate for sarcopenia remained imprecise and included the null value. Residual confounding and bidirectional relationships between hypertension, physical activity, comorbidity, body composition, and muscle health therefore remain possible (38).

Second, the number of available studies was limited, particularly for sarcopenic obesity and for subgroup-specific and cohort-restricted analyses. This limited the precision and statistical power of secondary analyses. Although random-effects models account for between-study variability in estimation, they do not resolve clinical or methodological heterogeneity. Accordingly, the absence of statistically significant subgroup differences should not be interpreted as evidence of equivalence between study designs, age groups, diagnostic approaches, or hypertension definitions. The borderline age subgroup finding for sarcopenic obesity should likewise be regarded as exploratory.

Third, methodological variation across the included studies limited comparability. Definitions and measurement approaches for sarcopenia, sarcopenic obesity, obesity, and hypertension were not uniform, and adjustment strategies differed across studies. Furthermore, the meta-analysis relied on aggregate study-level data, which prevented retrospective harmonization of exposure definitions and adjustment models and precluded detailed assessment of individual-level interactions among age, sex, adiposity, muscle function, and other cardiometabolic characteristics. Residual confounding by physical activity, diet, medication use, socioeconomic status, comorbidity burden, genetic susceptibility, and other unmeasured factors cannot be excluded. In addition, the included studies reported different relative effect measures across different observational designs. Although exploratory analyses stratified by effect measure type did not identify statistically significant subgroup differences, the small number of HR-reporting studies and the partial overlap between effect measure type, study design, and outcome timing limited the ability of these analyses to resolve effect-measure heterogeneity.

Fourth, the geographic distribution of the evidence limits generalizability. Most included studies were conducted in Asian populations, particularly in China and Korea, whereas evidence from other geographic and ethnic populations was comparatively limited. Differences in body composition, dietary patterns, physical activity, socioeconomic conditions, healthcare access, and hypertension management may influence the magnitude of the observed associations. Larger multicenter prospective studies involving diverse populations are needed to determine the consistency of these associations across clinical and geographic settings.

Finally, publication bias and other small-study effects cannot be excluded. Fewer than 10 studies contributed to each exposure-specific meta-analysis, limiting the statistical power of funnel plot inspection and Egger’s regression tests. Consequently, the absence of marked funnel plot asymmetry or statistically significant Egger’s test results should not be interpreted as evidence that publication bias is absent.

## 5 Conclusion

This meta-analysis showed that sarcopenia and sarcopenic obesity were significantly associated with hypertension, with pooled relative effect estimates of 1.45 and 2.49, respectively. The numerically larger estimate for sarcopenic obesity should not be interpreted as evidence of interaction or synergy because the two exposure-specific estimates were not formally compared. Given the observational nature of the evidence, substantial between-study heterogeneity, and the limited number of available studies, causal inference remains inappropriate. Further well-designed prospective studies using standardized definitions and diverse populations are needed to clarify temporality, sources of heterogeneity, and the clinical value of incorporating muscle health and adiposity into cardiometabolic assessment.

## Data Availability

The original contributions presented in the study are included in the article/Supplementary material, further inquiries can be directed to the corresponding author.
