# Supplementary material for: Associations of sarcopenia and sarcopenic obesity with hypertension: a systematic review and meta-analysis
Source: Front Nutr. 2026 Sep 7;13:1881526. doi: 10.3389/fnut.2026.1881526 (PMC13599406; doi:10.3389/fnut.2026.1881526)
Supplement: Supplementary file 1 [file Table_1.docx]

**Supplementary Table S1.** Complete database-specific search strategies and search results

**Search date: July 20, 2026**

PubMed:

| **No.** | **Query** | **Results** |
| --- | --- | --- |
| #1 | **Sarcopenia[MeSH Terms]** | 15,372 |
| #2 | **(((((((Sarcopenias[Title/Abstract]) OR (Muscleweakness[Title/Abstract])) OR (Low muscle mass[Title/Abstract])) OR(Decreased muscle mass[Title/Abstract])) OR (Musclestrength[Title/Abstract])) OR (Muscle loss[Title/Abstract])) OR(Muscle depletion[Title/Abstract])) OR (Muscularatrophy[Title/Abstract])** | 77,242 |
| #3 | **#1 OR #2** | 88,225 |
| #4 | **sarcopenic obesity[MeSH Terms]** | 1,488 |
| #5 | #3 OR #4 | 88,371 |
| #6 | **Hypertension[MeSH Terms]** | 339,839 |
| #7 | **(((((Blood Pressure, High[Title/Abstract]) OR (Blood Pressures,High[Title/Abstract])) OR (High Blood Pressure[Title/Abstract])) OR(High Blood Pressures[Title/Abstract])) OR (Elevated bloodpressure[Title/Abstract])) OR (Arterial hypertension[Title/Abstract])** | 81,124 |
| #8 | **#6 OR #7** | 374,139 |
| #9 | **#5 AND #8** | 470 |

Embase:

| **No.** | **Query** | **Results** |
| --- | --- | --- |
| #1 | 'sarcopenia/exp | 49,713 |
| #2 | 'sarcopenias':ti,ab OR 'muscle weakness':ti,ab OR 'low muscle mass':ti,ab OR 'decreased muscle mass':ti,ab OR 'muscle strength':ti,ab OR 'muscle loss':ti,ab OR 'muscle depletion':ti,ab OR 'muscular atrophy':ti,ab | 120,290 |
| #3 | #1 OR #2 | 158,680 |
| #4 | 'sarcopenic obesity'/exp | 2,999 |
| #5 | #3 OR #4 | 158,680 |
| #6 | 'hypertension'/exp | 1,271,240 |
| #7 | 'blood pressure, high':ti,ab OR 'blood pressures, high':ti,ab OR 'high blood pressure':ti,ab OR 'high blood pressures':ti,ab OR 'elevated blood pressure':ti,ab OR 'arterial hypertension':ti,ab | 133,618 |
| #8 | #6 OR #7 | 1,314,430 |
| #9 | #5 AND #8 | 6,828 |

Cochrane Library:

| **No.** | **Query** | **Results** |
| --- | --- | --- |
| #1 | [mh "Sarcopenia"] OR [mh "sarcopenic obesity"] OR [mh "Muscle Weakness"] OR [mh "Muscular Atrophy"] | 2,523 |
| #2 | ("Sarcopenia" OR "Sarcopenias" OR "Muscle weakness" OR "Low muscle mass" OR "Decreased muscle mass" OR "Muscle strength" OR "Muscle loss" OR "Muscle depletion" OR "Muscular atrophy" OR "sarcopenic obesity"):ti,ab,kw | 33,633 |
| #3 | #1 OR #2 | 33,633 |
| #4 | [mh "Hypertension"] | 26,482 |
| #5 | ("Hypertension" OR "Blood Pressure, High" OR "Blood Pressures, High" OR "High Blood Pressure" OR "High Blood Pressures" OR "Elevated blood pressure" OR "Arterial hypertension"):ti,ab,kw | 84,012 |
| #6 | #4 OR #5 | 84,014 |
| #7 | #3 AND #6 | 615 |

Web of Science:

| **No.** | **Query** | **Results** |
| --- | --- | --- |
| #1 | TS=("Sarcopenia" OR "Sarcopenias" OR "Muscle weakness" OR "Low muscle mass" OR "Decreased muscle mass" OR "Muscle strength" OR "Muscle loss" OR "Muscle depletion" OR "Muscular atrophy" OR "sarcopenic obesity") | 90,008 |
| #2 | TS=("Hypertension" OR "Blood Pressure, High" OR "Blood Pressures, High" OR "High Blood Pressure" OR "High Blood Pressures" OR "Elevated blood pressure" OR "Arterial hypertension") | 374,422 |
| #3 | #1 AND #2 | 1,554 |

**Supplementary Figure S1.** Exploratory analysis stratified by effect measure type for the association between sarcopenia and hypertension.

**Supplementary Figure S2.** Exploratory analysis stratified by effect measure type for the association between sarcopenic obesity and hypertension.
